# Phosphorylation‐Dependent Stabilization of Collaborator of ARF (CARF) Suppresses Lymphoma Cell Proliferation

**DOI:** 10.1002/advs.202416687

**Published:** 2025-07-06

**Authors:** Li Qu, Zhuang Wei, Shuting Zhou, Xiaofan Zhang, Wenjun Zhang, Aibin Liang, Zhe Wang, Hongwei Xue

**Affiliations:** ^1^ Joint Center for Single Cell Biology School of Agriculture and Biology Shanghai Jiao Tong University Shanghai 200240 China; ^2^ Key Laboratory of Systems Biology CAS Center for Excellence in Molecular Cell Science Chinese Academy of Sciences Shanghai 200031 China; ^3^ Department of Hematology Tongji Hospital School of Medicine Tongji University Shanghai 200092 China; ^4^ Guangdong Laboratory for Lingnan Modern Agriculture Guangdong Basic Research Center of Excellence for Precise Breeding of Future Crops College of Agriculture South China Agricultural University Guangzhou 510642 China

**Keywords:** CARF (Collaborator of ARF), cell division, lymphoma, phosphorylation, precise therapeutics

## Abstract

Uncontrolled cell proliferation drives tumorigenesis and malignant progression, making cell cycle regulation a promising strategy for cancer therapy. Phosphorylation plays pivotal roles in cancer initiation and metastasis by regulating the cancer‐related proteins. Identifying key phosphorylation sites is essential for inhibiting tumor cell proliferation and optimizing therapy strategy. Here, this study **reveals** the strong association of oncogene Collaborator of ARF (CARF), a cell‐division regulator interacting with p53, with prognosis and survival of lymphoma patients through pan‐cancer analysis. In addition, this study finds that mammalian CARF shares homology with Kip‐Related Protein6 (KRP6), a cell cycle inhibitor from higher plant *Arabidopsis*. KRP6 is regulated by casein kinase1 via phosphorylation at serines 75 and 109, which are conservative in CARF at serines 316 and 356. Systemic assays conducted with various B‐cell lymphoma cell lines and a mouse xenograft model demonstrate that the non‐phosphorylation variant of CARF inhibited cell proliferation and lymphoma formation more effectively than wild‐type CARF, highlighting the crucial regulatory role of phosphorylation at these conserved sites in controlling B‐cell lymphoma cell proliferation. A similar suppressive effect is observed with plant KRP6, suggesting a cross‐species bioengineering application. These findings enlighten the application of phosphorylation‐modified proteins as therapeutic targets in precise lymphoma treatments.

## Graphical abstract

This study revealed that oncogene Collaborator of ARF (CARF) is closely related to B‐cell lymphoma tumorigenesis, and shares a conserved mechanism regulating protein stability with *Arabidopsis* Kip‐Related Protein6 (KRP6) through casein kinase 1‐mediated phosphorylation. Reduced phosphorylation led to the enhanced protein stability of CARF, resulting in significantly suppressed cell proliferation and lymphoma tumorigenesis.

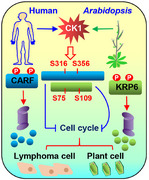

## 1 Introduction

Cell cycle is precisely regulated by cyclins, cyclin‐dependent kinases (CDKs), and checkpoint proteins.^[^ ^1^, ^2^ ^]^ Dysregulated cell proliferation, resulting from aberrant activity of various cell cycle regulators, is a hallmark of cancer cells and a driving force of tumorigenesis.^[^ ^2^, ^3^, ^4^, ^5^ ^]^ Consequently, targeting cell cycle regulators has emerged as a promising strategy for cancer therapy.^[^ ^2^ ^]^ B‐cell lymphomas are mainly caused by chromosomal translocations and genomic mutations in B cells and accounts for 95% of the lymphomas with aggressive and rapid progression, including Burkitt lymphoma (BL), high‐grade B‐cell lymphoma, NOS (HGBCL), or diffuse large B‐cell lymphoma (DLBCL).^[^ ^6^, ^7^ ^]^ Clinically, B‐cell lymphomas manifests as rapidly growing tumor involving one or more lymph nodes and extranodal sites due to elevated cell proliferation,^[^ ^8^ ^]^ which poses a serious threat to patient health and survival. Thus, identifying effective strategies to inhibit lymphoma cell proliferation and developing targeted therapies remain urgent and challenging tasks.

Protein phosphorylation is the most common and important post‐translational modification that regulates multiple biological processes, including tumorigenesis and cancer development by inducing the cancer cell proliferation, invasion, and metastasis as well as inhibiting the cancer cell apoptosis.^[^ ^9^, ^10^ ^]^ Recent advancements in functional profiling of phosphorylation sites (serine, threonine, and tyrosine) have revealed that mutations at these sites profoundly alter the function of target proteins involved in cell proliferation and tumor development,^[^ ^11^ ^]^ highlighting the immense therapeutic potential of phosphorylatable amino acids as molecular targets. Based on these phosphorylation sites, four major upstream kinase groups have been identified, including casein kinase 1 (CK1),^[^ ^11^ ^]^ a highly conserved serine/threonine kinase in eukaryotes that plays a crucial role in tumor progression through phosphorylation.^[^ ^12^ ^]^ CK1 family members have been shown to phosphorylate a wide array of substrates, including p53 and mouse double‐minute 2 homolog (MDM2).^[^ ^13^, ^14^, ^15^, ^16^ ^]^ A deeper understanding of CK1‐mediated phosphorylation mechanisms will not only advance our knowledge of cancer initiation and metastasis, but also facilitate the development of novel biomarkers and targeted therapeutic strategies.

Collaborator of ARF (alternative reading frame), CARF, has pleiotropic effects including regulation of DNA damage response, cell cycle checkpoints, and tumor suppression, primarily through modulation of the p53 pathway in both ARF‐dependent and ‐independent manners.^[^ ^17^, ^18^ ^]^ CARF functions as a multi‐module regulator, as attenuated *CARF* expression can trigger DNA damage response, leading to cell mitotic arrest and apoptosis. Conversely, overexpression of *CARF* inhibits cell proliferation and induces senescence via the p53‐HDM2‐p21 pathway.^[^ ^19^ ^]^ Notably, super‐expression of *CARF* could trigger pro‐proliferation through interaction with ERK,^[^ ^20^, ^21^ ^]^ suggesting that CARF may regulate cell proliferation by modulating its expression or protein stability. Additionally, treatment with a CARF‐targeting adenoviral vector resulted in a significant reduction in tumor size,^[^ ^19^ ^]^ underscoring CARF's potential as a therapeutic candidate for cancer therapy. However, the specific cancer types most responsive to *CARF* expression remain to be identified, which limits the potential clinical application of CARF‐based therapies for personalized tumor treatment.

In this study, we identified a robust correlation between *CARF* expression and diffuse large B‐cell lymphoma (DLBCL), as well as its close association with patient survival and enhanced sensitivity to Rituximab treatment in lymphoma patients. Interestingly, CARF is the human homolog of *Arabidopsis* Kip‐related protein 6 (KRP6), a cell cycle inhibitor, sharing conserved phosphorylation sites by CK1.^[^ ^22^ ^]^ The non‐phosphorylation variant of CARF dramatically inhibits cell proliferation in different lymphoma cell lines and mice xenograft tumor models in vivo, similar to non‐phosphorylation variant of KRP6, indicating that phosphorylation of CARF regulates cell proliferation and lymphoma tumorigenesis. Our studies provide new insights into the clinical and therapeutic potential of CARF phosphorylation sites for targeted lymphoma treatments and suggest that plant‐derived proteins may hold promise for disease diagnosis and precision therapy.

## 2 Results

### 2.1 *CARF* Expression is Strongly Correlated with Diffuse Large B‐Cell Lymphoma (DLBC)

**Figure 1:**
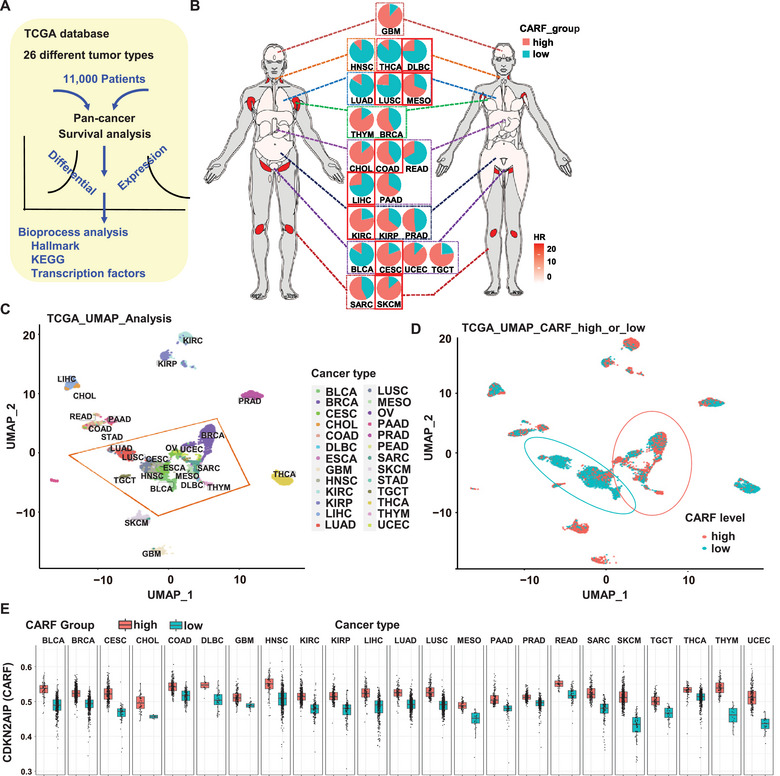
*CARF* expression is most strongly correlated with Diffuse Large B‐Cell lymphoma (DLBC). A) Analysis workflow of *CARF* expression and related cancer types. Data from 26 different cancer types in the TCGA database, comprising 11000 patients, were downloaded. The cutoff point of *CARF* gene expression and patient grouping for each cancer type was determined for survival analysis. Differential expression analysis of samples with different *CARF* gene expression was performed, and hazard ratios (HR) greater than “1” was identified in each cancer type. Differential genes were clustered, and enrichment analysis was conducted to identify the enriched biological processes or transcription factors corresponding to high or low *CARF* expression. B) Anatogram plot of HR for different cancer types grouped by high or low *CARF* expression. The color scale represents the increasing HRs with darker shades to indicating the higher risk. Male (left) and female (right) populations are shown. The central pie charts show the proportion of each tumor type grouped into high or low *CARF* expression (numbers and percentage of patients of each tumor type were shown in Table S1, Supporting Information), and cancer types exhibiting significant differences were highlighted by red boxes. Abbreviations: GBM, Glioblastoma Multiforme; HNSC, Head and Neck Squamous Cell Carcinoma; THCA, Thyroid Carcinoma; DLBC, Diffuse Large B‐Cell Lymphoma; LUAD, Lung Adenocarcinoma; LUSC, Lung Squamous Cell Carcinoma; MESO, Mesothelioma; THYM, Thymoma; BRCA, Breast Invasive Carcinoma; CHOL, Cholangiocarcinoma; COAD, Colon Adenocarcinoma; READ, Rectal Adenocarcinoma; LIHC, Liver Hepatocellular Carcinoma; PAAD, Pancreatic Adenocarcinoma; KIRC, Kidney Renal Clear Cell Carcinoma; KIRP, Kidney Renal Papillary Cell Carcinoma; PRAD, Prostate Adenocarcinoma; BLCA, Bladder Urothelial Carcinoma; CESC, Cervical Squamous Cell Carcinoma and Endocervical Adenocarcinoma; UCEC, Uterine Corpus Endometrial Carcinoma; TGCT, Testicular Germ Cell Tumors; SARC, Sarcoma; SKCM, Skin Cutaneous Melanoma. C) Clustering analysis of cancer types related to different *CARF* expression levels. The dimensionality reduction map, colored by different cancer types, was generated using UMAP (Uniform Manifold Approximation and Projection) based on gene expression levels with all cancer samples in TCGA database (https://doi.org/10.3389/fonc.2020.603702). UMAP1 and UMAP2 represent the two dimensions of projection. The topologically entangled clusters in the map correspond to cell types derived from mesoderm origin and epiblast with lower differentiation. D) Patient groups with different *CARF* expression levels on the U dimensionality reduction map. Dots and circles in red or blue represent the high or low *CARF* expression, respectively. E) Pan‐cancer expression status of *CARF* across different tumor types and *CARF* expression patient groups. The data points represent the *CARF* expression levels in individual patients.

To comprehensively understand the specific role of CARF in different tumors, we conducted a pan‐cancer‐level systematic analysis with the Cancer Genome Atlas Program (TCGA) database (**Figure** **1**A), which integrated data from approximately 11000 patient samples from 26 different cancer types.^[^ ^23^ ^]^ We determined the optimal cutoff for *CARF* expression in patient samples of each cancer type using the minimum *p‐*value approach and classified them into high and low groups, and summarized the percentage and number of patients of each tumor type (Table S1, Supporting Information). The results revealed that 8 cancer types (within red box in Figure 1B), including Diffuse Large B‐Cell lymphoma (DLBC), exhibited the most significant differences in patients with low *CARF* expression showing poorer prognosis compared to those with high *CARF* expression; while no significant differences were observed in other cancer types (Figure 1B; Figure S1, Supporting Information).

DLBC is the most prevalent subtype of non‐Hodgkin B‐cell lymphoma globally, accounting for approximately 30%–40% of all cases worldwide.^[^ ^6^ ^]^ Further dimensionality reduction analysis was performed, and the cancer type clusters with significant topological entanglement were primarily composed of cell types derived from mesoderm and epiblast origins (Figure 1C), suggesting that cells within these tumor types exhibit low differentiation and high similarity. Notably, diverse cancer types were clearly clustered, with the high and low *CARF* expression following distinct patterns (Figure 1D), indicating that CARF plays a key role in the development of less differentiated tumors. Pan‐cancer expression analysis was performed to assess the overall gene expression similarity across patients and their respective tumor types, and results further demonstrated the significant differences of *CARF* expression with DLBC (Figure 1E). Given that lymphoma is a type of hematological tumor, similar to leukemia, in which cells circulate freely, a reanalysis using a published single‐cell sequencing dataset for leukemia revealed that cell clusters exhibiting high *CARF* expression were enriched for genes related to cell cycle and DNA repair processes (Figure S2A–C, Supporting Information). In addition, a negative correlation was observed between the enrichment scores of DNA repair‐related genes and *CARF* expression (Figure S2D, Supporting Information). Considering that DNA repair process is closely related to p53 pathway and cell cycle arrest, these findings are consistent with the CARF functions in tumor regulation, particularly in controlling cell division.^[^ ^17^, ^18^ ^]^

Differential expression analysis and Gene Set Enrichment Analysis (GSEA) were conducted to explore the CARF‐associated biological processes. By analyzing the processes (cell cycle, inflammation, reactive oxygen species, changes in the p53 pathway) and marker genes (key oncogene *MYC*, tumor‐associated transcription factors) closely related to tumor progression,^[^ ^23^, ^24^, ^25^ ^]^ we surprisingly found an unexpected enrichment of most of these processes in the group of patients with low *CARF* expression (Figure S3, Supporting Information). Activation of p53 pathway is typically a stress response triggered by DNA damage or genomic instability, and enrichment of which suggests that cells under low CARF conditions may experience more damage and, as a compensatory mechanism, activate p53‐mediated repair and cell cycle arrest. In contrast, processes predominantly enriched in *CARF* high‐expression group were more closely related to benign physiological functions with a high degree of differentiation, including muscle formation and intercellular connection (Figure S3, Supporting Information). These results indicated that CARF plays a pivotal role in lymphoma tumorigenesis and its strong association with cell cycle and proliferation, suggesting that enhancing CARF protein stability could be an effective therapeutic strategy for lymphoma treatment.

### 2.2 *CARF* Expression is Essential for Lymphoma Patients’ Prognosis

**Figure 2:**
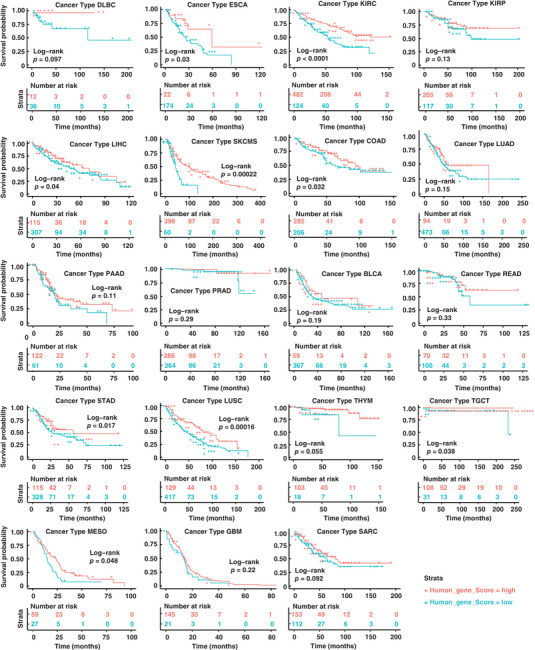
*CARF* expression is significantly associated with the survival of lymphoma patients. Survival curves analysis of *CARF* in different cancer types reveals a strong correlation between *CARF* expression and DLBC in TCGA database with an HR > 1. Abbreviations of cancer types are the same as in Figure 1B.

To investigate the correlation between *CARF* expression and patient prognosis, we performed a volcano plot analysis using data from TCGA database and result showed that high *CARF* expression was most strongly associated with a lower risk in lymphoma (DLBC), while thyroid cancer (THCA) showed the most significant association with poor prognosis (Figure S4, Supporting Information), consistent with the results of low hazard ratios (HR) of cancers with high *CARF* expression in Cox proportional hazards regression model analysis (Figure S5, Supporting Information). A good prognosis usually means a higher chance of survival, while a poor prognosis suggests a lower likelihood of survival. Further pan‐cancer and Kaplan‐Meier survival curve analyses indicated that high *CARF* expression was associated with better prognosis in the majority of cancer types (19 of 26), with particularly strong association in DLBC (HR > 1) (**Figure** **2**). Conversely, 7 types of cancer exhibited a poor prognosis associated with high *CARF* expression (HR <= 1, Figure S6, Supporting Information). To validate these findings in lymphoma, we reanalyzed the GSE10846 cohort with 420 patients^[^ ^26^ ^]^ considering the limited number of lymphoma samples in TCGA dataset. The result confirmed the significant correlation of high *CARF* expression with improved patient survival (HR = 1.78, conf int = 1.31–2.42) (Figures S7, and S8, Supporting Information). In addition, ARF, a participant in CARF function, exhibited the same trend (HR = 1.61, conf int = 1.15–2.27) (Figures S7, and S8, Supporting Information).

To assess the prognostic relevance of *CARF* in lymphoma, we further analyzed the additional tumor parameters, including mutation burden and chromosome structural variations.^[^ ^27^ ^]^ Results revealed that tumor patients with high *CARF* expression tend to have lower mutation burden, both for non‐silent mutations (*p *< 0.05) and silent mutations (*p *< 0.001) (Figure S9, Supporting Information). High *CARF* expression was also correlated with reduced chromosomal structural variation, including a lower Aneuploidy Score (*p *< 0.001) and a reduced percentage of genome doublings (Figure S9, Supporting Information), suggesting that *CARF* has a protective effect against tumor progression by stabilizing genome.

### 2.3 *CARF* Expression Closely Correlates with Rituximab Sensitivity in Lymphoma Clinical Treatment

To investigate the relationship between CARF expression and clinicopathological characteristics, a *chi*‐square test analysis was conducted and the result showed that no significant association between *CARF* expression and clinical factors such as patient age, gender, pathological subtype, Ann Arbor stage, Eastern Cooperative Oncology Group (ECOG) performance status, and Lactate dehydrogenase (LDH) levels (Table S2, Supporting Information). However, *CARF* expression was significantly associated with extranodal involvement and the chemotherapy regimen, particularly the treatment of cyclophosphamide, hydroxydaunorubicin, Oncovin, and prednisone (CHOP) regimen (*p* = 0.005661 and *p* = 1.28E‐29, respectively, Table S2, Supporting Information). Importantly, patients treated with an R‐CHOP‐Like regimen, which incorporates the CD20 monoclonal antibody Rituximab,^[^ ^28^ ^]^ exhibited significantly higher *CARF* expression (*p* = 0.005661) compared to those receiving conventional CHOP‐like chemotherapy (Table S2, Supporting Information), indicating that high *CARF* expression may have a strong correlation with high sensitivity to Rituximab in lymphoma treatment. Furthermore, patients with more than one extranodal sites also exhibited higher *CARF* expression (*p* = 0.005661), suggesting a potential association between high *CARF* expression and involvement of extralymphatic organs in cases of transfer‐related lymphoma with distant metastasis, although such cases comprised less than 10% of the cohort (Table S2, Supporting Information).

To examine whether high or low expression of *CARF* has a synergistic predictive effect on clinical prognosis with Rituximab treatment, a univariate analysis was conducted (Figure S9, Supporting Information) and results indicated that low *CARF* expression can predict poor patient prognosis (HR = 1.67, 95% CI = 1.23–2.27, *p* = 0.001, Table S2, Supporting Information). When comparing cases with low *CARF* expression and no Rituximab treatment to those with high *CARF* expression and Rituximab treatment, a significantly worse prognosis was observed (HR = 2.07, 95% CI = 1.44–2.96, *p *< 0.001). This interaction was designated as the Rituximab and CARF (R_CARF) indicator, which was validated as a prognostic predictor, alongside clinical factors in Table S2 (Supporting Information)^[^ ^29^ ^]^ (*p *< 0.01) (Figure S10, Supporting Information). To assess the prognostic predictive value of R_CARF indicator, highly significant variables from the univariate analysis were incorporated into a multivariate model, and results demonstrated that Rituximab treatment in conjunction with high *CARF* expression provided superior prognostic predictive ability (*p *< 0.001) compared to low *CARF* expression (Figure S11, Supporting Information). Moreover, several commonly used clinical indicators, like CHOP status, and ECOG performance, did not exhibit a level of significance comparable to that of the R_CARF indicator in predicting patient prognosis, and only LDH (*p *< 0.001) was at the similar level of predictive value (Figure S9, Supporting Information). However, R_CARF demonstrated greater clinical utility, as LDH could only serve as a prognostic marker, whereas R_CARF would also guide medication.

### 2.4 CARF is Homologous to *Arabidopsis KRP6* with Conserved Phosphorylation Sites

**Figure 3:**
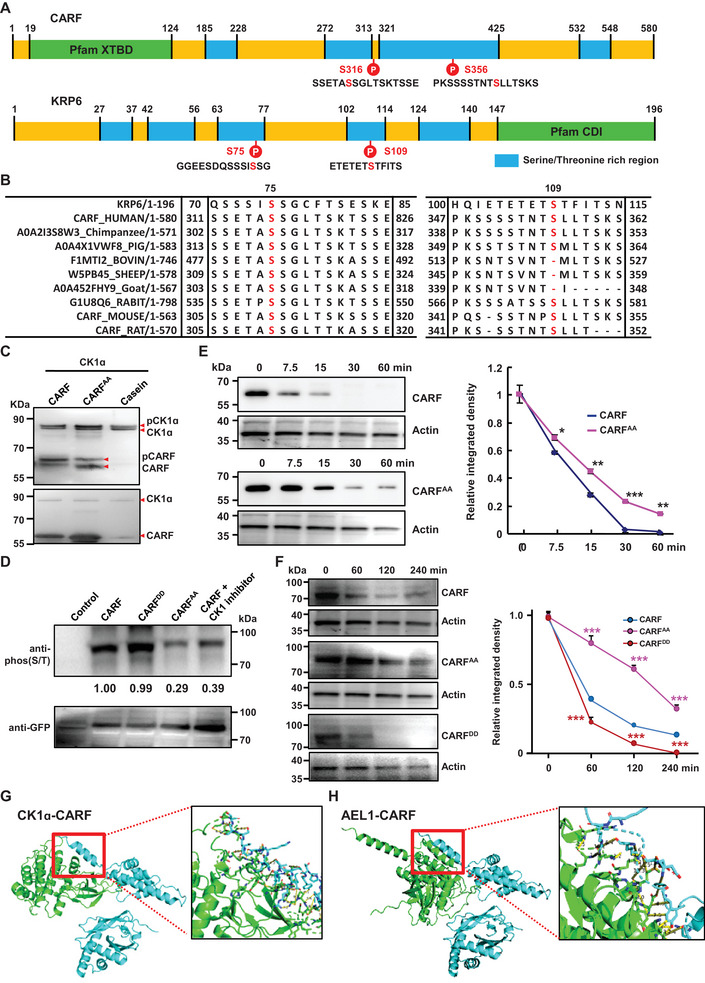
CARF is homologous to *Arabidopsis* KRP6 with conserved phosphorylation sites. A) Schematic illustration of CARF and KRP6 protein structures. Conserved phosphorylation sites by CK1 were shown. B) Multiple sequence alignment indicated that S75 and S109 of *Arabidopsis* KRP6 are highly conserved with S316 and S356 of CARF in human and animals across various species. C) Phos‐tag gel shift assay demonstrated that CK1α phosphorylates CARF and CARF^AA^ exhibited significantly reduced phosphorylation. Recombinant CK1α, CARF, and CARF^AA^ proteins were analyzed using an anti‐His antibody. Protein loading was confirmed by the presence of bands in gel (shown below). D) In vivo phosphorylation assay revealed that phosphorylation levels in cells expressing CARF^AA^ or CARF treated with CK1 inhibitor were significantly reduced. Burkitt's Ramos (RA1) lymphoma cells expressing EGFP‐fused CARF, CARF^AA^, and CARF^DD^ were used for analysis through immuno‐precipitation using GFP beads. Phosphorylation signals were detected using an anti‐phos (S/T) antibody, and protein loading was examined using an anti‐GFP antibody. Band quantities were calculated using Image J and relative band density ratio of phos (S/T)/GFP of CARF was defined as “1.00″. E) Western blotting analysis confirms the suppressed degradation of non‐phosphorylation variant CARF^AA^. The stability of purified Flag‐CARF and Flag‐CARF^AA^ fusion proteins was examined by in vitro cell‐free analysis using anti‐Flag antibody (left, Actin protein was analyzed with anti‐Actin antibody and used as loading control). Band density (right) was measured by Image J, with every relative band density normalized to the corresponding Actin bands. Relative density was calculated by setting normalized CARF/corresponding Actin (or CARF^AA^/corresponding Actin) at time 0 as 1.0. Data were means ± SD (n = 3), and statistical significance was determined by using one‐way ANOVA (*p < 0.05; **p < 0.01; ***p < 0.001). F) Western blotting analysis confirmed the in vivo suppressed degradation of non‐phosphorylation variant CARF^AA^ in RA1 transfected cells. The stability of CARF‐EGFP and CARF^AA^‐EGFP fusion proteins was examined using an anti‐GFP antibody (left, Actin levels were analyzed with anti‐Actin antibody and served as a loading control). The band density (right) was quantified using ImageJ, with every relative band density normalized to the corresponding Actin bands, and next relative density normalized CARF/corresponding Actin (or CARF^AA^/corresponding Actin) intensity at time 0, set as 1.0 for the degradation analysis. Data were means ± SD (n = 3), and statistical significance was determined by using one‐way ANOVA (*p < 0.05; **p < 0.01; ***p < 0.001). G,H) Structural docking model and interaction surface analysis of human CK1α (G) and *Arabidopsis* AEL1 (H) with CARF using HDOCK program. Proteins are shown in cartoon models (green for CK1α/AEL1, cyan for CARF). In the zoomed‐in images on right, the amino acids of CK1α/AEL1 form hydrogen bonds with CARF are displayed as stick models. Black font and yellow lines represent the hydrogen bonds and their lengths, as analyzed with PyMOL.

Protein structural analyses showed that CARF is the human homolog of *Arabidopsis* cyclin‐dependent kinase inhibitor Kip‐Related Protein 6 (KRP6, **Figure** **3**A). Our previous study demonstrated that *Arabidopsis* EL1‐like (AEL), a plant casein kinase 1 (CK1), regulates cell division by suppressing the stability of KRP6 through phosphorylation.^[^ ^22^ ^]^ Considering that CK1s are highly conserved in plants and mammals,^[^ ^32^ ^]^ further analysis revealed the phosphorylation sites Ser75 and Ser109 of KRP6 are conserved in CARF, corresponding to Ser316 and Ser356, respectively (Figure 3A,B), suggesting a post‐translational modification of CARF through phosphorylation.

To confirm whether human CK1 indeed phosphorylates CARF, the normal and non‐phosphomimic variant (of which the Ser316 and Ser356 were substituted with Alanine, abbreviated as CARF^AA^) of CARF was examined. A phos‐tag gel shift assay in vitro showed that CK1α phosphorylates CARF, while CARF^AA^ exhibited significantly reduced phosphorylation (Figure 3C). An in vivo phosphorylation assay further confirmed the reduced phosphorylation of CARF^AA^ by CK1α, as well as CARF in presence of CK1 inhibitor (Figure 3D), demonstrating that CK1α phosphorylates CARF at these two sites. Similarly to KRP6, in vitro cell‐free assays showed that compared to normal CARF, the non‐phosphomimic variant CARF^AA^ exhibited a suppressed degradation (Figure 3E), which is more significant by in vivo protein degradation assay, whereas the phosphomimic variant CARF^DD^ (of which the Ser316 and Ser356 were substituted with Aspartic acid, abbreviated as CARF^DD^) showed a much increased degradation (Figure 3F), demonstrating the crucial role of CK1‐mediated phosphorylation in reducing the CARF stability. In addition, protein‐protein docking using template‐based modeling and HDOCK program showed that the docking model of human CK1α with CARF (Figure 3G) is similar to that of AEL with CARF (Figure 3H), confirming the regulation of CARF by CK1 through phosphorylation at conserved sites.

### 2.5 Phosphorylation of CARF Regulates the Lymphoma Cell Proliferation

**Figure 4:**
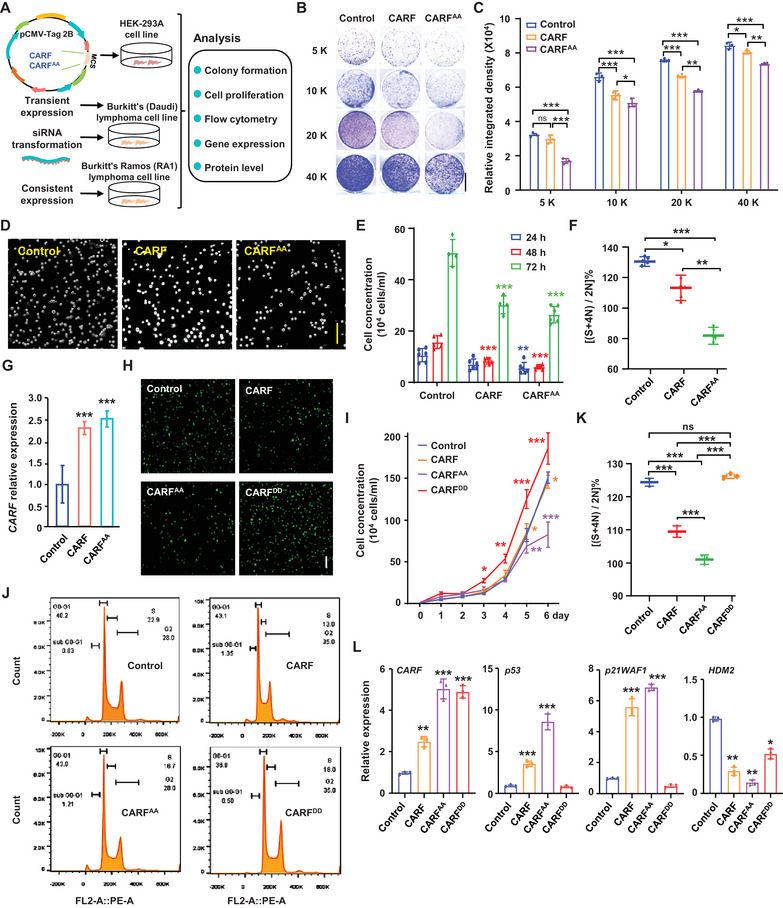
Phosphorylation of CARF plays key roles in regulating cell division. A) Flowchart illustrating the cell proliferation assay in HEK‐293A, Burkitt's Daudi, and Burkitt's Ramos (RA1) lymphoma cell lines. B,C) Cell proliferation assays revealed that CARF^AA^ significantly suppresses HEK‐293A cell division. Constructs containing CARF and CARF^AA^ were transfected into HEK‐293A cells. Cell growth was observed at initial densities of 5, 10, 20, or 40 K cells per well of 96‐well plate and cultured for 96 h after transfection (B, bar = 1 cm). Relative growth of cells was indicated by color, and darker colors correlate with higher cell density and better proliferation. Cell numbers per well were measured with relative density by Image J. Experiments were biologically repeated three times, and data were means ± SD (n = 3, C). Statistical significance was determined by using one‐way ANOVA (*p < 0.05; **p < 0.01; ***p < 0.001; ns, no significance). D,E) Cell proliferation assays revealed that CARF^AA^ suppresses cell division more significantly than CARF in Burkitt's Daudi lymphoma cell lines. Live cell concentration of Daudi lymphoma cells was analyzed at 24, 48, or 72 h post‐transfection, and growth of cells at 72 h was shown (D, bar =100 µm). Cell number was measured with cell counter. Experiments were biologically repeated three times and data were means ± SD (n = 6, E). Statistical significance was determined by using one‐way ANOVA (**p < 0.01; ***p < 0.001, compared to respective control transfection and highlighted with different colors). F) Flow cytometry analysis showed the suppression of CARF^AA^ on Daudi cell division. Transfected Daudi cells with constructs containing CARF or CARF^AA^ were digested into single cells after 96 h and cell division was analyzed by flow cytometry. “FL2‐A::PE‐A” represents a detection channel and the fluorescent dye used in the assay. Proportions of cells with different DNA content were calculated and data were means ± SD (n = 5). Statistical significance was determined by using one‐way ANOVA (*p < 0.05; **p < 0.01; ***p < 0.001, compared to vector transfection). G) qPCR analysis of *CARF* expression in transfection cells. Experiments were biologically repeated three times, and data were means ± SD (n = 3). Statistical significance was determined by using one‐way ANOVA (***p < 0.001, compared to vector transfection). H,I) Cell proliferation assay revealed that CARF^AA^ suppresses cell division more significantly than CARF in RA1 lymphoma cell lines. Constructs expressing *CARF*, *CARF^AA^*, and *CARF^DD^* were transfected into RA1 lymphoma cells respectively. Cell growth was observed at 0–6 days post‐transfection, and growth of cell at 6 days was shown (H, bar =100 µm). Cell number was measured with a cell counter. Experiments were biologically repeated three times, and data were means ± SD (n = 3, I). Statistical significance was determined by using one‐way ANOVA (*p < 0.05; **p < 0.01; ***p < 0.001, compared to vector transfection). J,K) Cytometry analysis showed that CARF^AA^ significantly suppresses cell division in RA1 lymphoma cells. Cells were digested into single cells at 96 h post‐transfection for cell division analysis by flow cytometry (J). Proportions of cells with different DNA content were calculated, and data were shown as means ± SD (n = 3, K). Statistical significance was determined by using Tukey's multiple comparisons test following one‐way ANOVA (***p < 0.001; ns, no significance). L) Expression of *CARF*, *p53*, *p21WAF1*, and *HDM2* in cells in (H) by qPCR analysis. Experiments were biologically repeated three times, and data were means ± SD (n = 3). Statistical significance was determined by using one‐way ANOVA (*p < 0.05; **p < 0.01; ***p < 0.001, compared to vector transfection).

To examine the effect of CARF phosphorylation in regulating lymphoma cell division, we performed a comprehensive analysis using HEK‐293A, Burkitt's (Daudi) and Burkitt's Ramos (RA1) lymphoma cell lines (**Figure** **4**A). Cell proliferation assays conducted in HEK‐293A cells transiently expressing *CARF* or non‐phosphomimic variant *CARF^AA^* showed that both CARF and CARF^AA^ profoundly inhibited cell proliferation and CARF^AA^ presented a more significant effect (Figure 4B,C), which is confirmed by cell proliferation assays and flow cytometry analysis in transiently transfected Burkitt's (Daudi) cells (Figure 4D–F) under a similarly upregulated transcriptional level (∼2.5 fold) of *CARF* and *CARF^AA^* (Figure 4G), indicating the enhanced effects of CARF^AA^ in suppressing cell proliferation due to the promoted stability resulting from reduced phosphorylation. In addition, silencing *CARF* in Burkitt's (Daudi) lymphoma cells by RNA interference (siRNA) showed a promoted cell division (Figure S12, Supporting Information), demonstrating that CARF functions mainly as a cell‐cycle inhibitor in lymphoma cells, which is consistent with the clinical observation that low *CARF* expression in lymphoma patients is associated with poorer prognosis and reduced survival.

We generated the Burkitt's Ramos (RA1) lymphoma cell lines expressing *CARF*, *CARF^AA^*, and phosphomimic variant *CARF^DD^* by the lentiviral delivery system with a co‐expressed *EGFP* reporter gene. Cell proliferation assay showed that CARF^DD^ promoted while both CARF and CARF^AA^ suppressed the cell division compared to control (Figure 4H,I). In addition, CARF^AA^ inhibited cell division more significantly compared to CARF, particularly at 5 or 6 days post‐transfection. Flow cytometry analysis revealed that compared to *CARF*, *CARF^AA^* reduced, while *CARF^DD^* increased, the cell proportion in S and M phase, and contrary tendency in G0/G1 phase (Figure 4J,K), confirming that CARF^AA^ profoundly inhibited RA1 cell proliferation. In addition, real‐time quantitative PCR (qPCR) and Western blotting analysis showed that the upregulated transcription and protein of *CARF* downstream targets *p53* and *p21WAF1*, and the suppressed transcription of downstream negative regulator *HDM2*, was more significant in *CARF^AA^*‐expressing cells (Figure 4L; Figure S13, Supporting Information). These results indicated that CARF^AA^ plays a more profound role and revealed the importance of phosphorylation of CARF in suppressing cell proliferation in lymphoma due to enhanced protein stability, and suggested their enormous potential being drug targets of effective therapeutic strategy for lymphoma.

### 2.6 Phosphorylation of CARF Regulates Cell Proliferation In Vivo

**Figure 5:**
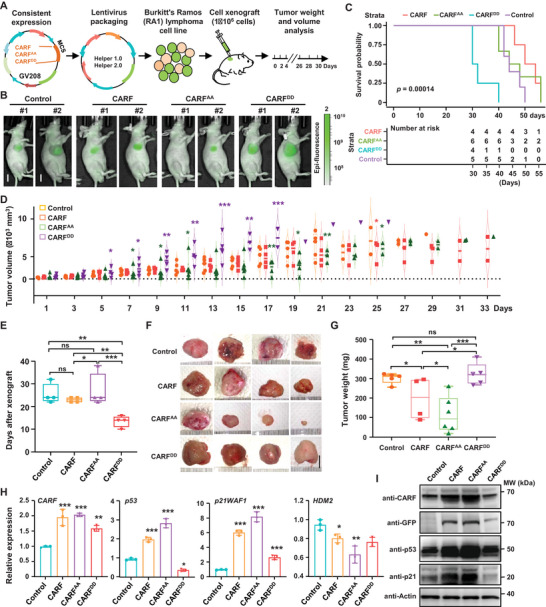
Phosphorylation of CARF regulates lymphoma cell division in a mice xenograft model. A) Schematic representation of the experimental design for investigating the roles of CARF and its phosphorylation variants in cell proliferation with Burkitt's Ramos (RA1) lymphoma cell and a xenograft model. B) Representative bioluminescent images showed the tumors in nude mice after subcutaneous injection of cells carrying vector (as control) or expressing *CARF*, *CARF^AA^*, and *CARF^DD^* (1× 10^6^ cells per mouse and 6 mice per group) for 28 days. Bar = 1 cm. C) Survival curves analysis showed the association of CARF phosphorylation sites with tumor progression in different mice xenografts. *CARF^AA^* showed improved, whereas *CARF^DD^* exhibited more aggressive, tumor progression, compared to mice xenografts overexpressing wild‐type *CARF*. D) Tumors growth curves in node mice after different cell xenografts. Tumor volumes were measured every 2 days post‐injection. Data were means ± SD (n = 4). Statistical significance was determined by using one‐way ANOVA (**p <0.01; ***p <0.001, compared to vector transfection). F)Analysis of the onset time of nodulation after different cell xenograft. Data were means ± SD (n = 4). Statistical significance was determined by using Tukey's multiple comparisons test following one‐way ANOVA (*p < 0.05; **p < 0.01; ***p < 0.001; ns, no significance). F,G) Tumor observation (F, bar = 1 cm) and tumor weight measurement (G) at the cutoff point. Data were means ± SD (n =5). Statistical significance was determined by using Tukey's multiple comparisons test following one‐way ANOVA (*p < 0.05; **p < 0.01; ***p < 0.001; ns, no significance). H) qPCR analysis of *CARF*, *p53*, *p21WAF1*, and *HDM2* gene expressions in tumor tissues from the xenograft model. Experiments were biologically repeated three times, and data were means ± SD (n = 3). Statistical significance was determined by using one‐way ANOVA (*p < 0.05; **p < 0.01; ***p < 0.001, compared to vector transfection). I) Western blotting analysis of CARF, p53, and p21 protein levels in tumor tissues from the xenograft model. Recombinant CARF protein or variants were co‐expressed with EGFP and examined using anti‐CARF and anti‐GFP antibodies. Proteins p53 and p21 were examined using anti‐p53 or anti‐p21 antibodies. Actin was used as a loading control.

To prove the role of phosphorylation modification of CARF in regulating cancer cells proliferation in vivo, we examined the antitumor effects of CARF and variants in lymphoma mice models. Mice xenograft experiments were conducted by injecting Burkitt's Ramos (RA1) lymphoma cells expressing *CARF* or variants into immune‐deficient mice for tumor formation, with consistent *GFP* gene expression for in vivo imaging of tumorigenesis in mice (**Figure** **5**A). GFP imaging showed that CARF, especially CARF^AA^ significantly reduced the tumor size at day 28 with delayed nodulation onset time after subcutaneous injection (Figure 5B). Analysis of tumor growth curves and volume measurements, as well as the survival curves, further indicated that CARF^AA^ significantly repressed while CARF^DD^ promoted the tumorigenesis than CARF (Figure 5C,D), similarly to the trend of nodulation onset time (Figure 5E) and tumor weight (Figure 5F,G). Considering that overexpressed *CARF* could impair proliferation by activating p53‐HDM2‐p21 pathway,^[^ ^17^, ^18^ ^]^ qPCR analysis revealed that tumors overexpressing *CARF^AA^* exhibited more significant upregulated expression of *p53* and *p21WAF1*, while the expression of downstream negative regulator *HDM2* was more reduced compared to *CARF‐*expressing tumors (Figure 5H), confirming the enhanced effect of CARF^AA^in tumor suppression. Western blotting analysis further showed the elevated protein levels of CARF, p53, and p21WAF1 proteins in tumor cells overexpressing *CARF* and *CARF^AA^* (Figure 5I), indicating the crucial roles of phosphorylation in stabilizing the CARF protein and modulating cell division in vivo.

### 2.7 Transcriptome Analysis Revealed the Important Role of Phosphorylation in CARF‐Mediated Regulation of Cell Division

**Figure 6:**
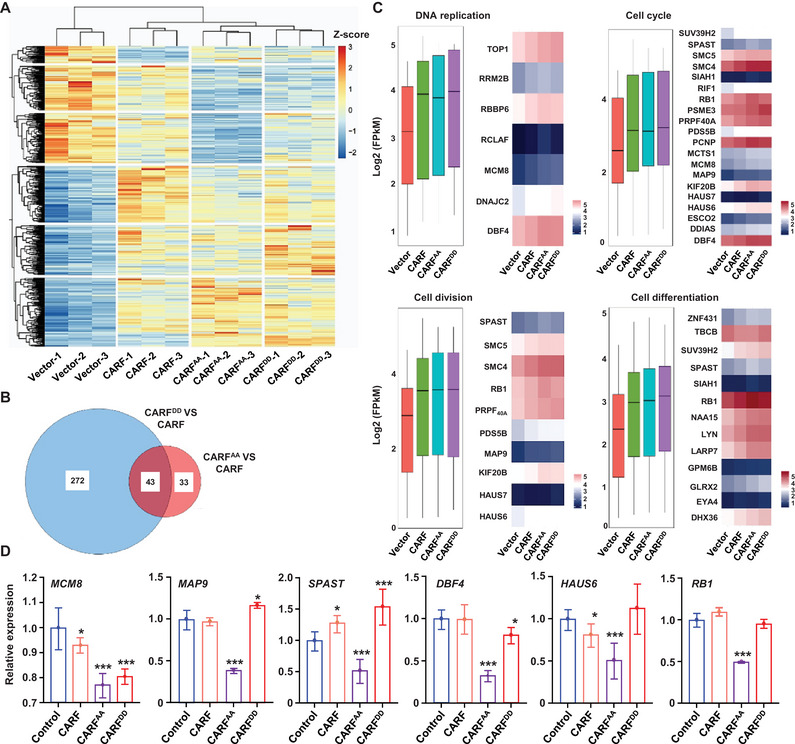
Transcriptome sequencing (RNA‐seq) analysis revealed the important role of phosphorylation of CARF in cell division regulation. A) Heatmap of all differentially expressed genes (DEGs) at 48 h after cell culture, with |log2FC| > 0.5, adjusted *p *< 0.05. RNA‐seq was conducted with RA1 cells expressing *CARF* and variants *CARF^AA^* and *CARF^DD^* at 0, 24, and 48 h post‐culture. Variations of DEGs distribution at 48 h between CARF and variants were shown. Experiments were biologically repeated three times. Z‐score is calculated with log2‐transformed FPKM values. The color gradient (right) reflects the gene expression levels. B) Venn diagrams representing the overlap of DEGs in RA1 cells expressing *CARF^AA^* (red) or *CARF^DD^* (blue) compared to *CARF* (|log2FC| > 0.5, adjusted *p *< 0.05). The central overlap (dark red) depicts the common DEGs. Experiments were biologically repeated three times. C) Boxplot and heatmap of expression of DEGs involving in DNA replication, cell cycle, cell division, and cell differentiation. The relative expression in the boxplot is calculated with log2‐transformed FPKM values. D) qPCR analysis of expressions of selected maker genes depicted in Figure 6C. Experiments were biologically repeated three times, and data were means ± SD (n = 3). Statistical significance was determined by using one‐way ANOVA (*p < 0.05; ***p < 0.001, compared to control).

To further elucidate the role of CARF phosphorylation in regulating cell division, we performed the transcriptome analysis using RNA‐sequencing (RNA‐seq) of RA1 cells expressing *CARF* and variants *CARF^AA^* and *CARF^DD^* at 0, 24, and 48 h post‐culture. Analysis of the differentially expressed genes (DEGs) (Figure S14, Supporting Information) revealed an increase in the number of DEGs at 48 h, with a significant variation in DEGs distribution between CARF and variants (**Figure** **6**A, and Figure S14, Supporting Information). Enrichment analysis of the 159 co‐upregulated and 179 co‐downregulated in *CARF^AA^* and *CARF^DD^* compared to *CARF* expressing cells (Figure S15, Supporting Information) suggested the involvement of both variants in regulating cell division signaling pathways are similar.

We identified 315 DEGs in *CARF^DD^* and76 DEGs in *CARF^AA^* compared to *CARF* expressing RA1 cells, and among them, 43 overlapped DEGs were common to both variants (Figure 6B). Gene Ontology (GO) enrichment and Kyoto Encyclopedia of Genes and Genomes (KEGG) pathway of the 43 DEGs showed a strong association with pathways related to DNA replication, cell cycle progression, cell division and cell differentiation (Figures S16,S17, Supporting Information), which are crucial for DNA replication and cell division and proliferation. In addition, the expression patterns of these DEGs correlated to cell cycle progression and cell division ability in different *CARF* expressing RA1 cells (Figure 6C). We further performed a qPCR analysis on these specific marker genes, particularly those overlapping across the biological processes including DNA replication, cell cycle/division/differentiation. Results confirmed the significant differences in expression of examined genes between CARF and mutants (CARF^AA^ and CARF^DD^) (Figure 6D), confirming that CARF phosphorylation plays an essential role in cell cycle through regulating DNA replication.

These findings are consistent with the single‐cell sequencing data from leukemia samples and the observed negative correlation between DNA repair‐related genes and *CARF* expression (Figures S2, and S3, Supporting Information). Enrichment analysis of molecular functions of the 43 DEGs further highlighted the pivotal roles of phosphorylation in cell cycle related process including DNA replication, microtubule‐based process, and protein transport and localization (Figure S18, Supporting Information).

### 2.8 Non‐Phosphorylation Variant of *Arabidopsis* KRP6 Inhibits Lymphoma Formation and Growth

**Figure 7:**
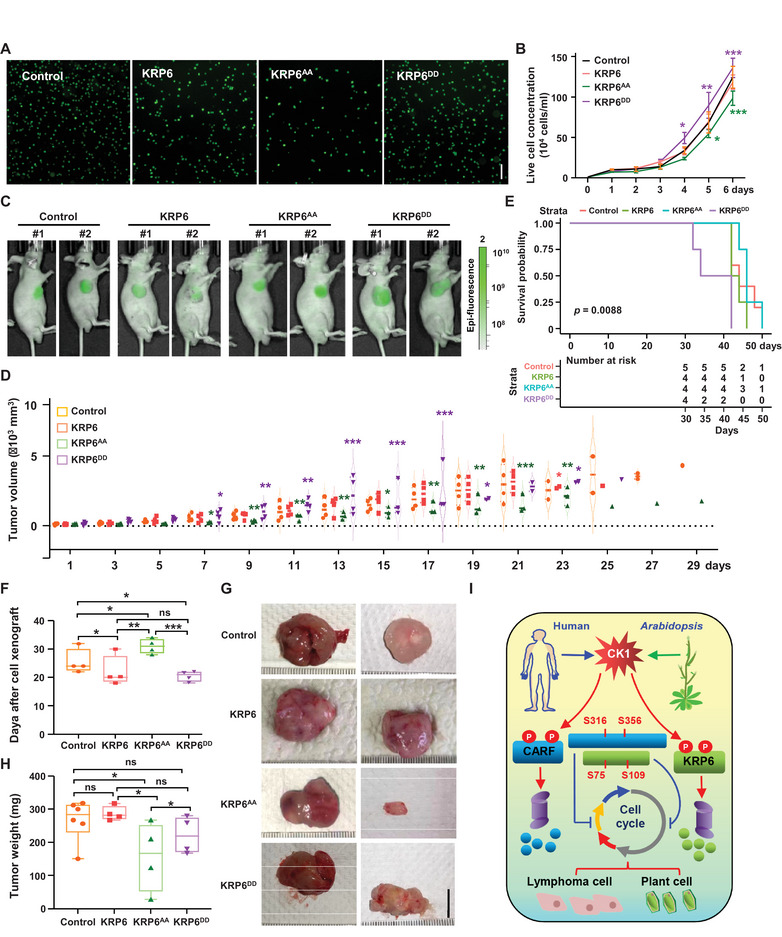
*Arabidopsis* KRP6 inhibits lymphoma formation and growth. A,B) Cell proliferation assays revealed that KRP6^AA^ suppresses cell division more significantly than KRP6 in RA1 lymphoma cells (A). Cell growth was observed at 24, 48, and 72 h post‐transfection. Cell number was measured with a cell counter. Experiments were biologically repeated three times, and data were means ± SD (n = 3, B). Statistical significance was determined by using one‐way ANOVA (*p < 0.05; **p < 0.01; ***p < 0.001, compared to vector transfection). C) Representative bioluminescent images showed the tumors in nude mice after subcutaneous injection of RA1 lymphoma cells carrying vector (as control) or expressing *KRP6*, *KRP6^AA^* and *KRP6^DD^* (1 × 10^6^ cells per mouse and 6 mice per group) for 28 days. Bar = 1 cm. D) Tumor growth curves in nude mice after different cell xenografts. Tumor volumes were measured every 2 days post‐injection. Data were means ± SD (n = 3). Statistical significance was determined by using one‐way ANOVA (**p < 0.01; ***p < 0.001, compared to vector transfection). E) Survival curves analysis showed the association of KRP6 phosphorylation sites with tumor progression in different mice xenografts. *KRP6^AA^* showed improved, whereas *KRP6^DD^* exhibited more aggressive, tumor progression, compared to mice xenografts overexpressing wild‐type *KRP6*. F) Analysis of the onset time of nodulation after different cell xenografts. Data were means ± SD (n = 4). Statistical significance was determined by using Tukey's multiple comparisons test following one‐way ANOVA (*p < 0.05; **p < 0.01; ***p < 0.001; ns, no significance). G,H) Tumor observation (G, bar = 1 cm) and tumor weight measurement (H) at 28 days post‐injection. Data were means ± SD (n = 5). Statistical significance was determined by using Tukey's multiple comparisons test following one‐way ANOVA (*p < 0.05; **p < 0.01; ***p < 0.001; ns, no significance). I) A hypothetical model of conserved CK1 function in regulating division of both plant and human cells by destabilizing KRP6 or CARF through phosphorylation.

Given the conservation of CARF and *Arabidopsis* KRP6, as well as the conserved phosphorylation sites, we examined the effects of plant KRP6 and variants on mammalian cells. The *KRP6* gene was cloned from *Arabidopsis*, and the phosphorylation sites Ser75 and Ser109 were mutated to Alanine (abbreviated as KRP6^AA^) or to Aspartic acid (abbreviated as KRP6^DD^). *KRP6* and variants were overexpressed in RA1 lymphoma cells (Figure S19A, Supporting Information). As expected, cell proliferation assay revealed that KRP6^AA^ (mimicking non‐phosphorylation) dramatically inhibited RA1 lymphoma cell proliferation (Figure 7A,B), suggesting that plant‐derived KRP6 protein can interfere with cell‐cycle in mammalian cells, and may serve as a potential exogenous lymphoma suppressor. Flow cytometry analysis demonstrated that *KRP6^AA^* expression significantly decreased the proportion of cells in S and M phases, while *KRP6^DD^* had the opposite effect to increase the cell proportion in these phases. Conversely, there was an increased proportion of G0/G1 phase in *KRP6^AA^*‐expressing cells compared to those expressing *KRP6^DD^* (Figure S19B, Supporting Information), confirming that KRP6^AA^ profoundly inhibited RA1 cell proliferation. qPCR and Western blotting analysis revealed the upregulated expression and protein levels of *p53* and *p21WAF1*, and suppressed expression of the negative regulator HDM2, were more significant in *KRP6^AA^*‐expressing cells (Figure S19A,C, Supporting Information). Interestingly, we also observed an increased RNA level of *p21WAF1* and decrease RNA level of *HDM2* upon KRP6^DD^ expression, suggesting that plant‐derived KRP6 protein has the capacity to interfere with the cell‐cycle machinery in mammalian cells by a different mechanism from CARF, and that KRP6 may functions as a potential exogenous suppressor of lymphoma. Further examination by in vivo phosphorylation assay showed a reduced phosphorylation of KRP6^AA^ by CK1α, as well as KRP6 in presence of CK1 inhibitor (Figure S19D, Supporting Information). Consistently, in vivo protein stability assay revealed that KRP6^AA^ exhibited a suppressed protein degradation (Figure S19E, Supporting Information). These results highlighted the critical roles of KRP6 and its phosphorylation in suppressing cell proliferation in lymphoma.

Analysis using a mice xenograft model, in which RA1 cells expressing *KRP6* and its variants were injected into immune‐deficient mice for tumor formation, showed that KRP6, particularly KRP6^AA^, substantially inhibited the tumorigenesis and development (Figure 7C), which was confirmed by the repressed tumor growth curves and reduced tumor volume increases (Figure 7D), promoted survival curves (Figure 7E), delayed nodulation onset time (Figure 7F) and decreased tumor size (Figure 7G) and tumor weight (Figure 7H), similarly to the effect of CARF^AA^. As expected, KRP6^DD^ showed contrasting effects on tumor development and enhanced tumorigenesis, and accelerated tumor growth.

## 3 Discussion

Precision medicine in oncology relies on a comprehensive understanding of the correlation between specific proteins and tumor types. By integrating a pan‐cancer systematic analysis with various public databases, we identified a strong association between CARF and lymphoma tumorigenesis, patient survival, and Rituximab treatment. CARF is a homolog of plant cell cycle inhibitor KRP6, and we further demonstrated that the CK1‐mediated phosphorylation affects protein levels of CARF, which is crucial for proliferative potential of lymphoma cancer cells. Our finding underscores the significance of phosphorylation in controlling cell proliferation through modulating protein stability (Figure 7I), providing informative insights into the importance of CARF in lymphoma progression and therapeutic inventions.

Protein phosphorylation is crucial for tumorigenesis and development and has been successfully targeted in therapeutic strategies, particularly with kinase inhibitors,^[^ ^30^ ^]^ such as tyrosine kinase inhibitors in lymphomas treatment.^[^ ^31^ ^]^ However, the therapeutic approaches targeting substrates or specific phosphorylation sites are still rare. Recent studies highlighted the critical role of phosphorylation sites in regulating cell proliferation and tumor progression.^[^ ^11^ ^]^ Based on the strong correlation between *CARF* expression and lymphoma, we demonstrated that the phosphorylation of CARF at S316 and S356 is integral to tumorigenesis. The marked suppression of cell proliferation by the non‐phosphorylation variant of CARF indicates the potential of modulating CARF phosphorylation as a strategy for lymphoma therapy and suggests that these phosphorylation sites could be viable targets for lymphoma‐specific drugs, shedding light on the phosphorylation‐based application in lymphoma therapy at the amino acid level.

Both protein structural organization and function of CK1s are highly conserved across eukaryotes.^[^ ^32^ ^]^ Members of ubiquitously expressed and pleiotropic CK1 family play essential regulatory roles in a wide spectrum of cellular functions such as cell cycle progression and developmental regulation.^[^ ^12^, ^22^, ^32^ ^]^ Despite various studies elucidating the importance of CK1s in regulating cell division,^[^ ^12^, ^13^, ^14^, ^15^, ^16^, ^22^ ^]^ the precise regulatory mechanism and development of specific inhibitors for personalized cancer therapy remain challenging. The reduced CK1α expression has been observed in lymphomas compared to respective benign tissue, while the underlying mechanisms remain unclear.^[^ ^33^ ^]^ Our studies establish a strong link between CK1 and lymphoma, through phosphorylating CARF, illustrating a potential role for CK1‐mediated phosphorylation in regulating lymphoma. Our study offers new insights into the potential drug targets for lymphoma therapy from the perspective of substrate at amino acid level rather than CK1 itself, which is an important step toward advancing CK1‐mediated phosphorylation and phosphorylation sites of substrates to medical applications more precisely, considering the pleiotropic functions of CK1.

Notably, CK1 phosphorylates both plant KRP6 and human CARF at conserved phosphorylation sites to regulate cell division suggest that the mechanisms by which CK1 regulates cell division may share commonalities between plants and humans, highlighting the evolutionary functional conservation of CK1 across species and implying that insights gained from studying CK1 in one system may be applicable to understanding its function in another. However, there are also some inconsistencies in RNA levels of key cell cycle regulators in cells expressing KRP6 compare to CARF and their mutants, indicating the differences in regulatory mechanisms of KRP6 and CARF and underscoring the complexity of CK1‐mediated regulations.

Cross‐species bioengineering has shown the promising results in both basic research and translational medicine. Recent studies demonstrated that the ectopic expression of plant RDR1 protein in cancer cell lines achieved a broad‐spectrum anti‐tumor response.^[^ ^34^ ^]^ Conversely, the expression of an animal obesity gene, *FTO*, in rice and potato significantly improved yield as well.^[^ ^35^ ^]^ In our study, ectopic expression of plant KRP6 protein in lymphoma cells resulted in significant inhibition of cell proliferation, which provides an example for cross‐species bioengineering application and the promising application of plant proteins as anti‐lymphoma agents, indicating the important therapeutic efficacy of plant protein in animal cells. Although KRP6 shows tumor‐specific suppression, its effects on normal hematopoietic cells remain unexplored, representing a limitation of this study. Future investigations on this aspect will help to assess the KRP6's therapeutic potential. Our findings will expand and enhance the understanding of protein phosphorylation in lymphoma progression and guide the development of novel, more specific therapeutic strategies. In addition, plant proteins like KRP6, affect tumor cell proliferation underscores their potential as innovative therapeutic agents, contributing to expanding the horizon of cancer treatment strategies, offering new possibilities for developing alternative agents derived from plant proteins.

## 4 Conclusion 

Cell proliferation is a tightly regulated process influenced by a variety of factors and is integral to numerous physiological functions. We here revealed that *CARF* expression is closely related to B‐cell lymphoma tumorigenesis, lymphoma patient prognosis, and sensitivity to Rituximab treatment. As the human homolog of *Arabidopsis* KRP6, CARF shares a conserved regulatory mechanism through phosphorylation‐mediated protein stability. We verified that the non‐phosphorylation variants of both CARF and KRP6 effectively suppress cell proliferation and inhibit lymphoma tumorigenesis, revealing the crucial role of phosphorylation in regulating CARF and KRP6 function in the context of lymphoma. Our studies demonstrate the potential for modulating CARF phosphorylation as a clinical‐therapeutic strategy and suggest the promising application of plant‐derived proteins as anti‐lymphoma drugs, shedding light on more precise and targeted approaches to lymphoma treatment and providing insights for cross‐species bioengineering application.

## 5 Experimental Section

### Cell Culture, Plasmids, and Transfection

HEK‐293A cells and Burkitt's (Daudi) lymphoma cells were obtained from the American Type Culture Collection (ATCC, Manassas, VI, USA). Burkitt's Ramos (RA1) lymphoma cells were purchased from GENE (Australia). For the constructs used in transient transfection, Flag‐tagged CARF and KRP6 were amplified and subcloned into pCMV‐tag2B vector. Resultant constructs were used to generate single or double amino acids mutation (CARF^AA^, CARF^DD^, KRP6^AA^, and KRP6^DD^) with Fast Mutagenesis System (FM111‐01, TransGen Biotech, China). In stable transfection, all constructs were delivered via the lentiviral system to either culture or generate mice xenograft model. Cells were cultured in Dulbecco's modified Eagle's medium (DMEM) supplemented with 10% fetal bovine serum (FBS) at 37°C with 5% CO_2_. Transfection was performed with Lipofectamine 3000 (Invitrogen, USA) following manufacture's protocol. Used primers were listed in Table S3 (Supporting Information).

### Expression and Purification of Recombinant Proteins From E. coli

Recombinant CARF‐His and CARF^AA^‐His proteins were expressed in *E. coli* strain BL21 (DE3). The open reading frames (ORFs) of genes were amplified and subcloned into pET51b vector (His tag, Novagen, Germany), and resultant constructs were used to generate the double amino acids mutation with Fast Mutagenesis System (FM111‐01, TransGen Biotech).

### In Vitro Protein Degradation Assay by Cell‐Free Analysis

Cell‐free assay was performed according to previous description.^[^ ^36^ ^]^ Equal amounts of HEK‐293A cells were harvested and ground into fine powder in liquid nitrogen. Total proteins were extracted using a buffer consisting of 25 mM Tris·HCl (pH 7.5), 10 mm NaCl, 10 mm MgCl_2_, 4 mm PMSF, 5 mm DTT, and 10 mm ATP. Cell debris was removed by twice centrifugations (12,000 rpm, 10 min, and 4 °C) to collect the supernatants. Protein concentration was determined by Pierce BCA Protein Assay Kit (ThermoFisher Scientific, USA). Purified CARF‐His and CARF^AA^‐His proteins were incubated in total protein extracts (150 mL, 500 mg) at 22 °C. Protein abundances were determined by immunoblot using anti‐His‐HRP‐conjugated antibody (M20020L, 1:5000, Abmart, China).

### In Vivo Degradation Assay

Stable transfectants of RA1 cells expressing CARF‐EGFP, KRP6‐EGFP, or variant proteins were collected. After two days of incubation, cells were treated with medium containing 3 µm MG132 for 12 h to pre‐accumulate protein. Cells were then incubated in medium supplemented with 50 µm cycloheximide (CHX, C7698, Sigma‐Aldrich) to inhibit protein synthesis for different time points (0, 30, 60, 120, or 240 min), then harvested and immediately frozen in liquid nitrogen. Proteins were extracted with extraction buffer (PEB, 20 mm Tris‐HCl, pH 7.5, 150 mm NaCl, 0.5% Tween‐20, 1 mm EDTA, 1 mM DTT) containing a protease inhibitor cocktail (Complete, 0 469 311 6001, Roche) and phosphatase inhibitor cocktail (PhosSTOP, 04906845001, Roche). The abundance of proteins was determined by western blotting using anti‐GFP antibody (ab290, Abcam, UK).

### Structure Docking Modeling

The protein structures of AEL1, CK1α, and CARF were obtained from Protein Data Bank (PDB). Protein‐protein docking was performed with the HDOCK algorithm,^[^ ^37^ ^]^ and resultant models were visualized and analyzed in the PyMOL program.^[^ ^38^ ^]^

### Cell Proliferation Assay

HEK‐293A cells were seeded in 6‐well plates and cultured to 80%–90% confluence by day 2. Transfections were carried out using 1.5 µg plasmid DNA per well and Lipo3000 reagent (Invitrogen). For proliferation assay, transfected cells were plated in 96‐well plates at four different initial seeding densities (5, 10, 20, and 40 K cells per well) and cultured for 4 days. After incubation, the medium was removed and cells were fixed and stained with 0.1% crystal violet in formaldehyde for 15 min. Plates were then imaged, and cell numbers were quantified by measuring the relative staining intensity using ImageJ. After washing with PBS, excess dye was removed by rinsing with water, and images were captured after air dying. A similar assay was conducted with Daudi lymphoma cells and lentivirus delivery system was used to obtain the stable transfected Burkitt's Ramos (RA1) lymphoma cells.

### Flow Cytometry Analysis

For cell proliferation analysis with flow cytometry, after cells were transfected for 6 h, the medium was replaced and cells were digested with trypsin A (100 µg mL^−1^) for 30 min, and propidium iodide (50 µg mL^−1^) was added and incubated in dark for 30 min. Cell analysis was performed using a FACSCelesta Flow cytometer (BD company, USA), and data were analyzed using Flow J software.

### Real‐Time Quantitative PCR (qPCR) Assay

Total RNA was extracted from cell samples using Trizol reagent (Cat#15596026, Invitrogen) according to the manufacturer's instructions. First‐strand cDNA was reverse transcribed from 1 µg of total RNA using the PrimeScript 1st Strand cDNA Synthesis Kit (TaKaRa, Japan). qPCR was performed with the SYBR Premix Ex Taq (Cat#QPK‐201, Toyobo, Japan) on the Bio‐Rad real‐time PCR system. Data were analyzed by the comparative CT method, with Actin used as the internal control for normalization. Used primers are listed in Table S3 (Supporting Information).

### Western Blotting Analysis

Burkitt's Ramos (RA1) lymphoma cells or tumor tissues were grounded into powder and extracted with Protein Extraction Buffer (PEB, SB‐BR040, Share‐bio, China). Protein samples were separated by 10% sodium dodecyl sulfate‐polyacrylamide gel electrophoresis (SDS‐PAGE) gel (Genescript, China), then transferred onto polyvinylidene difluoride membranes (Perkin‐Elmer, USA). Proteins were detected using anti‐CARF (70R‐16334, Fitzgerald), anti‐GFP (ab290, Abcam, UK), anti‐p53 (ab26, Abcam), anti‐p21 (ab109520, Abcam), and anti‐Beta Actin (ab8226, Abcam) antibodies, followed by HRP‐conjugated goat anti‐rat IgG secondary antibodies (AP202P, 1:10000, Sigma, Germany). HRP activity was detected using SuperSignal western detection reagents (Share‐bio, China) and Chemiluminescence imaging system (Tanon 4800, China).

### Phos‐Tag Gel Shift Assay and In Vitro Phosphorylation

Protein samples were incubated with or without CK1α kinase in an in vitro phosphorylation reaction. After reaction, proteins were separated on a Phos‐tag gel. Phosphorylated proteins exhibit slower migration compared to non‐phosphorylated counterparts. Proteins were then analyzed using an anti‐His antibody (M20020, Abmart, America). Protein loading was confirmed by presence of bands corresponding to the target protein.

### In Vivo Phosphorylation Assay

RA1 cells stably transfected with CARF, KRP6, or variants were incubated for 3 days, then collected. CK1 inhibitor was CK1α 1 blocking peptide (33R‐3833, Fitzgerald, America). Proteins were immunoprecipitated with GFP beads (M20016, Abmart, America) and phosphorylation signals were examined with anti‐phos (S/T) antibody (ab17464, Abcam, UK). Loading of proteins was examined with an anti‐GFP antibody (ab290, Abcam, UK).

### CARF siRNA Knockdown and Transfection

For knockdown of CARF, 21‐nucelotide RNAs were synthesized using Expedite RNA phosphoramidites and thymidine phosphoramidite (Sangon, Shanghai). Synthetic oligonucleotides were deprotected and gel purified. Sequences of control and two target oligos for CARF were listed in Table S3 (Supporting Information). SiRNAs were annealed by incubating 20 µM single strands in annealing buffer (100 mm KAC, 30 mm HEPES‐KOH at pH 7.4, 2 mm MgAC) for 1 min at 90 °C followed by 1 h at 37 °C. Transfections of siRNA duplexes were carried out using Oligofectamine reagent (Life Technologies). Of the 20 µM duplexes, 1–5 µL were used per 12‐well dish and assayed after 24–48 h by qPCR.

### In Vivo Tumor Models

Burkitt's Ramos (RA1) lymphoma cells expressing CARF, CARF^AA^, CARF^DD^, KRP6, KRP6^AA^, and KRP6^DD^ were obtained via lentiviral delivery to generate mice xenograft model by injecting into BALB/c nude mice subcutaneously with ≈10^6^ cells per mouse. Tumor volume was measured with calipers, and tumors were harvested when tumor volume in control mice reached >7500 mm^3^.

### In Vivo Imaging System

Cell imaging was performed using the Invitrogen EVOS FL Auto Cell Imaging System. Mice and tumors images were acquired and analyzed using PE IVIS Spectrum system and Living Image 4.7.2 software (Caliper Life Sciences, USA). Data visualization and statistical analysis were conducted using GraphPad Prism9.

### Grouping Analysis

Grouping analysis was conducted according to previous description.^[^ ^39^ ^]^ All tumor cases from TCGA and the lymphoma cohort GSE10846 were grouped and analyzed according to CARF expression levels using a recursive algorithm that recursively split the data into smaller subsets to maximize the log‐rank test statistic. Finally, the patients were classified into high and low CARF expression groups.

### Kaplan‐Meier Survival Curve Analysis

The analysis refers to the previous description.^[^ ^40^ ^]^ Specifically, all cases from TCGA and the lymphoma cohort GSE10846 were analyzed by survival curves according to tumor types. Survival time was calculated based on enrollment and event times. The survival probability at each time point was determined by dividing the number of patients without an event by the total number of patients, excluding those lost to follow‐up.

### Determination of Optimal Cutoff Based on Minimum p‐Value Approach

To classify tumor patients into “low‐risk” and “high‐risk” groups based on gene expression levels, a statistical method utilizing survival analysis was employed to determine the optimal cutoff point for a continuous gene expression variable. Specifically, this method considers each possible value of gene expression as a candidate cutoff, divides patients into two groups accordingly, and calculates the significance of survival curve differences between the groups using the Log‐rank test. The cutoff point that maximizes the inter‐group difference was selected as the optimal threshold. This cutoff effectively separates patients into two groups with significant prognostic differences, facilitating the subsequent survival analysis and risk assessment, thereby uncovering the potential biological significance of gene expression level in patient prognosis. The code for this analysis refers to the previous paper.^[^ ^41^ ^]^

### Cox Proportional Hazards Regression Model Analysis and Univariate Analysis and Multivariate Analysis

Cox regression and multivariate analyses were conducted as previously described.^[^ ^42^ ^]^ Briefly, the CARF high and low expression groups, along with other clinicopathological factors, were analyzed using a Cox proportional hazards regression model. Univariate analysis performed regression analysis on each single factor to obtain hazard ratios (HR). Among the results obtained by univariate analysis, significant ones were selected for multivariate analysis. Multivariate analysis used maximum partial likelihood estimation (MLE) to calculate coefficients and corresponding standard errors for each covariate. The HR was the ratio of the coefficients of the two covariates.

### Pathway and Functional Annotation and GSEA Analysis

Gene set enrichment analysis (GSEA) was performed on TCGA clinical data to identify biological processes associated with CARF‐high or ‐low expression cases, based on the known gene signature database that has been published and summarized in previous studies.^[^ ^43^ ^]^ The required known gene signature database uses the Explore the Molecular Signatures Database (MSigDB) database, specifically the C1, C2 KEGG, and C3 TF datasets.^[^ ^44^ ^]^ The normalized enrichment score (NES) was plotted in R.

### Single‐Cell mRNA Sequencing Data Analysis

Single‐cell mRNA sequencing data from acute leukemia (AML) GSE110499^[^ ^45^ ^]^ were reduced dimension by tSNE method using Monocle package provided in R.^[^ ^46^, ^47^ ^]^ The gene sets of DNA repair and cell cycle were obtained from CancerSEA database^[^ ^48^ ^]^ and enrichment scores were calculated using the GSVA package in R.^[^ ^49^ ^]^ Correlation analysis was performed using the Pearson method.

### RNA Extraction and Sequencing Analysis

RNA of Burkitt's Ramos (RA1) lymphoma cell lines expressing *CARF*, *CARF^AA^*, and *CARF^DD^* after 0, 24, and 48 h was extracted using TRIzol® Reagent with three biologically replicates. RNA purity and quantification, and RNA integrity were evaluated using NanoDrop 2000 spectrophotometer (Thermo Fisher Scientific) and Agilent 2100 Bioanalyzer (Agilent Technologies, USA), respectively. Samples with RNA integrity number (RIN) 7.0 were subjected to sequencing analysis, and the libraries were constructed with ABclonal mRNA‐seq Lib Prep Kit (ABclonal, China) and TruSeq Stranded mRNA LTSample Prep Kit (Illumina) after sequencing on the Illumina Novaseq 6000 /MGISEQ‐T7 platform. Sequencing data were processed using an in‐house bioinformatics pipeline from Shanghai Applied Protein Technology.

### Sequence Analysis of Homologous Proteins and Phosphorylation Sites

Homologous analysis between plant KRP6 and mammalian CARF proteins was performed using uniprot website (https://www.uniprot.org/) and Amino acid sequence tools (http://www.aminode.org/gene=CDKN2AIP). Protein sequences for KRP family members were obtained from TAIR (https://www.arabidopsis.org/). Multiple protein alignments to identify conserved phosphorylation sites were conducted by MAFFT. Protein structure analysis was conducted by using SMART (https://smart.embl‐heidelberg.de/). Protein phosphorylation sites were predicted using Scansite4.0 website (https://scansite4.mit.edu).

### Software and Database

The TCGA datasets were downloaded from TCGA website (https://www.cancer.gov/tcga.), cBioPortal (http://www.cbioportal.org/),^[^ ^50^ ^]^ and UCSC Xena (http://www.xena.ucsc.edu/).^[^ ^51^ ^]^ Dataset GSE10846, a publicly available gene expression profiling dataset that comprises gene expression data and clinical information for 414 non‐Hodgkin lymphoma (NHL) patients, was downloaded from the Gene Expression Omnibus (GEO) database (https://www.ncbi.nlm.nih.gov/geo/) for analysis.

### Animal Care

All animal studies were approved by the Institutional Animal Care and Use Committee of Shanghai Jiao Tong University (Experimental Animal Study Protocol No. A2022042) and conducted following institutional guidelines. All animals (mice) were housed in small groups in a specific pathogen‐free facility with a 12 h day/night cycle and had ad libitum access to food and water. Animals were acclimated for at least 1 week following their arrival to the facility prior to each study.

## Conflict Interest

The authors declare no conflict of interests.

## Author Contributions

L.Q. and H.X. designed experiments. L.Q., Z.W., S.Z., W.Z., and A.L. conducted the experiments. L.Q., Z.W., and X.Z. analyzed and interpreted the data, and drafted the manuscript. L.Q. and H.X. wrote the paper.

## Supporting information



Supporting Information

## Data Availability

The data that support the findings of this study are available from the corresponding author upon reasonable request.
