# Supplementary material for: Phosphorylation‐Dependent Stabilization of Collaborator of ARF (CARF) Suppresses Lymphoma Cell Proliferation
Source: Adv Sci (Weinh). 2025 Jul 6;12(33):e16687. doi: 10.1002/advs.202416687 (PMC12412514; doi:10.1002/advs.202416687)
Supplement: Supplementary file 1 — Supporting Information [file ADVS-12-e16687-s001.pdf]

ADVANCED SCIENCE
Open Access

Supporting Information

for *Adv. Sci.*, DOI 10.1002/advs.202416687

Phosphorylation-Dependent Stabilization of Collaborator of ARF (CARF) Suppresses Lymphoma Cell Proliferation

*Li Qu, Zhuang Wei, Shuting Zhou, Xiaofan Zhang, Wenjun Zhang, Aibin Liang, Zhe Wang and Hongwei Xue**

Supplementary Material includes 19 figures and 3 tables, and is available online.

Supplementary figure legends.

Figure S1. Anatogram plot of hazard ratios (HRs) for different tumor types grouped by high or low *CARF* expression, excluding DLBC. The color gradient reflects increasing HRs, with darker shades indicating higher risk for both male (left) and female (right) groups. The central pie chart shows the distribution of each tumor type categorized by high or low *CARF* expression. The abbreviations of cancer types are the same as in Figure 1B.

Figure S2. *CARF* expression is associated with p53-related DNA repair and cell cycle regulation and inversely correlated with DNA repair in single-cell dataset.
A-C. TSNE dimensionality reduction map of GSE110499 single-cell mRNA sequencing dataset from acute leukemia (AML). Color scale of points is GSVA enrichment score for DNA repair (A), cell cycle (B), or the normalized *CARF* mRNA expression values (C). tSNE1 or tSNE2 indicated two dimensions of projection.
D. Correlation analysis showed that *CARF* expression is closely related to DNA repair score. R is the correlation coefficient and p is the significance.

Figure S3. Biological processes enriched in tumor patients with high or low *CARF* expressions from TCGA database.
Gene Set Enrichment Analysis (GSEA) of hallmark genes and *CARF* expression. Mutsig C1 Hallmark dataset (A), Kyoto Encyclopedia of Genes and Genomes (KEGG) dataset (B), and Mutsig C3 transcription factor dataset (C) are shown. Bars represent the Normalized Enrichment Score (NES), quantifying the strength and direction of gene set enrichment.
D. Volcano Plot illustrates the correlation between CARF expression and GSVA enrichment scores for gene sets in Lymphoma (GSE10946). Each dot represents a gene set from the Mutsig database related to cell proliferation, cell cycle, or p53 pathway. The x-axis shows the Pearson correlation between CARF expression and gene set's GSVA score. Gene sets meeting significance ($p < 0.05$ and a preset correlation threshold) are highlighted and labeled. Positively correlated gene sets are mainly linked to cell proliferation and cell cycle, whereas negatively correlated ones are associated with p53 pathway, which is consistent with that higher *CARF* expression is associated with increased proliferation and reduced p53 activity

in lymphoma.

Figure S4. Volcano plot shows the differential *CARF* expression in TCGA database. The *x*-axis represents log2 fold change, indicating the magnitude of gene expression differences, and *y*-axis shows the log2(*p*-value), reflecting the statistical significance. The hyperbolic curve serves as a dynamic threshold that balances the statistical significance and biological relevance. Genes above the curve meet the criteria for both sufficient expression change and robust statistical significance, and are considered significantly differentially expressed. This method ensures the selection of genes with meaningful biological and statistical impact, reducing noise and avoiding the arbitrary cutoff biases.

Figure S5. Volcano plots show the survival analysis of high or low *CARF* expression in different cancers with (A) or without (B) DLBC. Hazard Ratio (*x*-axis) and *p*-value (*y*-axis) for high and low *CARF* expression with (A) and without (B) DLBC were shown.

Figure S6. A pan-cancer analysis of *CARF* gene in TCGA database with HR <= 1. Survival curves for high and low *CARF* expression in different cancers with HR <= 1.

Figure S7. Grouping and survival analysis of *ARF* (A, *CDKN2A*) and *CARF* (B, *CDKN2AIP*) in Lymphoma GSE1084 database show that *CARF* is closely related to lymphoma. High and low expression groups were defined based on the minimum *p*-value cutoff, and final cutoffs optimized through result-oriented methods. Expression level distributions (middle) and standardized Log-Rank Statistic scatter plots (bottom) are shown. Standardized Log-Rank Statistic is a measure used in survival analysis, showing the probability of survival or the occurrence of events in individuals over a certain period of time.

Figure S8. Survival analysis of *CARF* or *ARF* transcription levels in lymphoma patients. Cancer prognosis analysis showed a strong correlation between *CARF* or *ARF* transcription levels and prognosis in lymphoma patients. Low (indicated by green) or high (indicated by red) *CARF* expression was associated with worse or better prognosis, as determined using the SurvivaX tool (http://bioinformatica.mty.itesm.mx:8080/Biomatec/SurvivaX.jsp) and the Lenz Staudt Lymphoma GSE10846 database including prognostic information of 420

patients.

Figure S9. Analysis of *CARF* gene in Lymphoma GSE1084 database.

A. Aneuploidy Score, a metric for chromosomal abnormalities in a sample, indicates the extent of aneuploidy.

B. Genome doublings, representing the occurrence of chromosomal duplication in a cell or organism.

C. TMB non-silent, the number of non-synonymous mutations per megabase in a tumor genome, indicates the presence of functional alterations ($p < 0.05$).

D. TMB silent, the number of synonymous mutations per megabase in a tumor genome, indicates no functional alteration ($p < 0.001$).

Figure S10. Univariate analysis of *CARF* expression and clinicopathological features in lymphoma GSE10846 cohort.

Figure S11. Multivariate analysis of *CARF* expression groups and clinicopathological features in lymphoma GSE10846 cohort.

Figure S12. siRNA of *CARF* promotes cell division in Burkitt's (Daudi) lymphoma cell lines.

A. Visualization of tagged CARF-siRNA-GFP in Burkitt's (Daudi) lymphoma cells shows that *CARF*-siRNA promotes cell division. Bar = 100 μm.

B. qPCR analysis of *CARF* expression in different siRNA transfection lines. Experiments were biologically repeated three times and data were means ± SD (n = 3). Statistical significance is determined by using one-way ANOVA (***, $p < 0.001$, compared with vector transfection).

C. Live cell concentrations of cell lines at 0, 24, 48 or 72 h post-transfection. Experiments were biologically repeated three times and data were means ± SD (n = 5). Statistical significance is determined by using one-way ANOVA (*, $p < 0.05$; **, $p < 0.01$; ***, $p < 0.001$, compared with vector transfection).

Figure S13. Western blotting analysis of CARF, p53 and p21 proteins in RA1 lymphoma cell lines 6 days post-transfection. Constructs expressing *CARF*, $CARF^{AA}$ and

CARF^{DD} were transfected into RA1 lymphoma cell lines, respectively. Actin was used as a loading control.

Figure S14. Heatmap of the differentially expressed genes (DEGs) from RNA-seq analysis.

Heatmap of all DEGs in transcriptome sequencing (RNA-seq) analysis with $|\log2FC| > 0.5$, adjusted $p < 0.05$. RNA of RA1 lymphoma cells expressing *CARF*, *CARF*^{AA}, *CARF*^{DD} at 0, 24 and 48 h were extracted and used for sequencing. Experiments were biologically repeated three times. Z-score was calculated with log2-transformed FPKM values.

Figure S15. RNA-seq analysis shows the differential gene expression in CARF variants with mutated phosphorylation sites.

A. Venn diagrams representing the overlapped upregulated and downregulated genes in RA1 cells expressing *CARF*^{AA} or *CARF*^{DD} compared to CARF ($|\log2FC| > 0.5$, adjusted $p < 0.05$). Experiments were biologically repeated for three times.
B. Distribution and number of DEGs in RA1 cells expressing *CARF*^{AA} or *CARF*^{DD} compared to *CARF* or vector ($|\log2FC| > 0.5$, adjusted $p < 0.05$).

Figure S16. **Functional enrichment analysis of DEGs related to DNA replication regulation.** Size of dots reflects the number of genes.

Figure S17. **Molecular functions of DEGs in the transcriptome sequencing analysis.** The top 25 GO categories are shown in descending order (right) with statistical significance ($|\log2FC| > 0.5$, adjusted $p < 0.05$).

Figure S18. Molecular function analysis of DEGs shows the crucial roles of phosphorylation sites of CARF in cell cycle regulation.

Enrichment analysis of molecular functions of DEGs in RA1 cells expressing *CARF*^{AA} or *CARF*^{DD} compared to *CARF* revealed the enriched cell cycle regulation related processes including DNA replication, microtubule-based process and protein transport and localization.

Figure S19. Non-phosphorylation variant of *Arabidopsis* KRP6 inhibits lymphoma

formation and growth.

A. qPCR analysis of *KRP6*, *P53*, *P21WAF1*, and *HDM2* expression in RA1 transfection cells. Experiments were biologically repeated three times, and data were means ± SD (n = 3). Statistical significance was determined by using one-way ANOVA (*, $p < 0.05$; **, $p < 0.01$; ***, $p < 0.001$, compared to vector transfection).

B. Cytometry analysis showed that $KRP6^{AA}$ significantly suppresses the cell division in RA1 lymphoma cells. Cells were digested into single cells at 96 h post-transfection for cell division analysis by flow cytometry (left). "FL2-A::PE-A" represents a detection channel and the fluorescent dye used in the assay. Proportions of cells with different DNA content were calculated and data were shown as means ± SD (n = 3, right). Statistical significance was analyzed by using Tukey's multiple comparisons test following one-way ANOVA (*, $p < 0.05$; **, $p < 0.01$; ***, $p < 0.001$; ns, no significance).

C. Western blotting analysis of KRP6, p53 and p21 protein levels in RA1 transfection cells. Recombinant KRP6 protein or variants were co-expressed with EGFP and examined using anti-GFP antibodies. Proteins p53 and p21 were examined using anti-p53 or anti-p21 antibodies. Actin was used as a loading control.

D. *In vivo* phosphorylation assay showed that phosphorylation levels of cells expressing $KRP6^{AA}$ or *KRP6* with CK1 inhibitor reduced significantly. Burkitt's Ramos (RA1) lymphoma cells expressing *KRP6*, $KRP6^{AA}$ and $KRP6^{DD}$ fused with EGFP were used. Anti-phos (S/T) indicates the phosphorylation signals and anti-GFP antibody confirms the protein loading.

E. Western blotting analysis confirms the suppressed degradation of non-phosphorylation variant $KRP6^{AA}$ in RA1 transfection cells. Stability of KRP6-EGFP and $KRP6^{AA}$-EGFP fusion proteins was examined with anti-GFP antibody (left, Actin protein was analyzed with anti-Actin antibody and used as loading control). Band density (right) was measured by Image J, and relative density was normalized to KRP6 (or $KRP6^{AA}$) intensity at time 0, which was set as 1.0. Data were means ± SD (n = 3), and statistical significance was determined by using one-way ANOVA (**, $p< 0.01$; ***, $p < 0.001$).

Table S1. The percentage and number of patient samples for each cancer type in Figure 1B and Figure S1.

Cancer type	*CARF*_group			
	High		Low	
	Percentage (%)	No.	Percentage (%)	No.
GBM	88.6	147	11.4	19
HNSC	11.7	66	88.3	500
THCA	12.8	73	87.2	499
DLBC	25	12	75	46
LUAD	15.8	91	84.2	485
LUSC	23.7	131	76.3	421
MESO	69	60	31	27
THYM	85.2	104	14.8	18
BRCA	57.2	695	42.8	520
CHOL	86.7	39	13.3	6
COAD	58.9	192	41.1	134
READ	33.7	35	66.3	69
LIHC	26.7	113	73.3	310
PAAD	66.1	121	33.9	62
KIRC	78.5	476	21.5	130
KIRP	64.1	207	35.9	116
PRAD	51.6	267	48.4	250
BLCA	14.1	60	85.9	367
CESC	81.2	251	18.8	58
UCEC	87.9	167	12.1	23
TGCT	77	107	23	32
SARC	57.4	152	42.6	113
SKCM	86.5	409	13.5	64

Table S2. Relationship between high or low *CARF* expression groups and clinicopathological features in lymphoma GSE10846 cohort.

Characteristic	No.	*CARF*_group		χ^2	*P value*
		high	low		
***CARF*_group**				420	2.44E-93
high	259	259	0		
low	161	0	161		
**Age_group**				2.39899784	0.121413
Age > 60 yr	179	117	62		
Age ≥ 60 yr	235	136	99		
**Gender**				0.1294861	0.718965
female	172	106	66		
male	224	142	82		
**Pathological_type**				2.665681863	0.263727
Unclassified DLBCL	64	37	27		
GCB DLBCL	183	106	77		
ABC DLBCL	167	110	57		
**Ann_Arbor_stage**				1.041066524	0.307574
Ann Arbor stage = I	66	37	29		
Ann Arbor stage > II	354	222	132		
**CHOP_status**				127.744813	1.28E-29
R-CHOP-Like Regimen	233	198	35		
CHOP-Like Regimen	181	55	126		
**Extranodal_sites**				7.655101087	0.005661
extranodal_sites < = 1	353	203	150		
extranodal_sites > 1	30	25	5		
**ECOG_performance**				1.04642836	0.306331
ECOG_performance < = 1	296	180	116		
ECOG_performance > 1	93	51	42		
**LDH**				3.86E-07	0.999504
LDH < = 1X	173	104	69		
LDH > 1X normal	178	107	71		

Table S3. Sequences of primers used in this study. Added restriction enzymes are underlined and mutated bases for site-directed phosphorylation mutations are double underlined. Non-phosphorylation or phosphorylation mimicking mutation is with the change of Serine (S) to Alanine (A) or to Aspartic acid (D), respectively.

Name	Sequence (5'-3')	
CARF-LP	CGCGGATCCATGGCGCAGGAGGTGTCGGAG	*BamH I*
CARF-RP	ACGCGTCGACTAACCCACTTGAAGCTGTCTCTG	*Sal I*
CARF-Flag-LP	GGAATTCATGGCGCAGGAGGTGTCGGAG	*EcoR I*
CARF-Flag-RP	CGCGGATCCTAACCCACTTGAAGCTGTCTCTG	*BamH I*
CARF-S316A-LP	TAGTTCAGAGACAGCTGCTAGTGGGTTAACTTCCA	
CARF-S316A-RP	TGGAAGTTAACCCACTAGCAGCTGTCTCTGAACTA	
CARF-S356A-LP	CTCTTCAACAAATACAGCTCTGCTAACTTCCAAGA	
CARF-S356A-RP	TCTTGGAAGTTAGCAGAGCTGTATTTGTTGAAGAG	
CARF-BD-LP	CATGGAGGCCGAATTCATGGCGCAGGAGGTGTCGGAG	*EcoR I*
CARF-BD-RP	GCCGCTGCAGGTCGACTAACCCACTTGAAGCTGTCTCTG	*Sal I*
CARF-S316D-LP	TAGTTCAGAGACAGCTGATAGTGGGTTAACTTCCA	
CARF-S316D-RP	TGGAAGTTAACCCACTATCAGCTGTCTCTGAACTA	
CARF-S356D-LP	CTCTTCAACAAATACAGATCTGCTAACTTCCAAGA	
CARF-S356D-RP	TCTTGGAAGTTAGCAGATCTGTATTTGTTGAAGAG	
CK1a-AD-LP	TGGAGGCCAGTGAATTCATGGCGCAGGAGGTGTCGGAG	*EcoR I*
CK1a-AD-RP	TCGAGCTCGATGGATCCTTACTGATAGGAAGGATAAAG	*BamH I*
CK1a-51B-LP	CGGATCCGACGTCGGTCGACATGGCTCTGCTGTCCGAGG	*Sal I*
CK1a-51B-RP	GCTCCTGCGGCCGCAAGCTTCTGATAGGAAGGATAAAGT	*Hind III*
CARF-51B-LP	CGGATCCGACGTCGGTCGACATGGCGCAGGAGGTGTCGG	*Sal I*
CARF-51B-RP	GCTCCTGCGGCCGC AAGCTTCCCACTTGAAGCTGTCTCTG	*Hind III*
CARF-siRNA-LP	CGGAGUACCUGAGCCAGAAUT	
CARF-siRNA-RP	UUCUGGCUCAGGUACUCCGUT	
RT-GAPDH-LP	AGGTCGGAGTCAACGGATTTG	
RT-GAPDH-RP	TGTAAACCATGTAGTTGAGGTCA	
RT-CARF-LP	TCAAAGTGACAGATGCTCCA	
RT-CARF-RP	CGTTGAACTGTTTTCCTGCT	
RT-HDM2-LP	GAGATATGTTGTGAAAGA	

RT-HDM2-RP	AATGGCATTAAGGGGCAAAC	
RT-p53-LP	CTGCCCTCAACAAGATGTTTTG	
RT-p53-RP	CTATCTGAGCAGCGCTCATGG	
RT-p21WAF1-LP	ATGAAATTCACCCCCTTTCC	
RT-p21WAF1-RP	CCCTAGGCTGTGCTCACTTC	
RT-Actin-LP	CTGGAACGGTGAAGGTGACA	
RT-Actin-RP	CTAAGGGACTTCCTGTAACAATGCA	
siRNA-NC-LP	AAGACCGAGUCCAUGAGGCUT	
siRNA-NC-RP	GCCUCAUGGACUCGGUCUUUT	
CARF-siRNA-LP	CGGAGUACCUGAGCCAGAAUT	
CARF-siRNA-RP	UUCUGGCUCAGGUACUCCGUT	
RT-KRP6-LP	ACCAGCAATTTCAGAAAAGAGACG	
RT-KRP6-RP	ACCTTCAAGCGGTTCGTCAT	
RT-MCM8-LP	TGGCTATGCTCGGCAGTATG	
RT-MCM8-RP	GCTGCCTGGTAGTGATTGGT	
RT-DBF4-LP	CCTCCCGAAGACGCTGTTT	
RT-DBF4-RP	TATGCTGAGCACCAGCGAAC	
RT-MAP9-LP	GGCGCTAACTGCATCCACTA	
RT-MAP9-RP	CTGCGGCGGTTGAAGATTTG	
RT-SPAST-LP	ATGAATTCTCCGGGTGGACG	
RT-SPAST-RP	CAGAGCCACACGAAGAGGAG	
RT-RB1-LP	GCGCTCAGTTGCCGGG	
RT-RB1-RP	CTCCCGACTCCCGTTACA	
RT-HAUS6-LP	GTCACCGCTTTCGAGAAGGA	
RT-HAUS6-RP	GGGGCCAACAAAATACTCCG	

Qu et al., Supp Figure 1

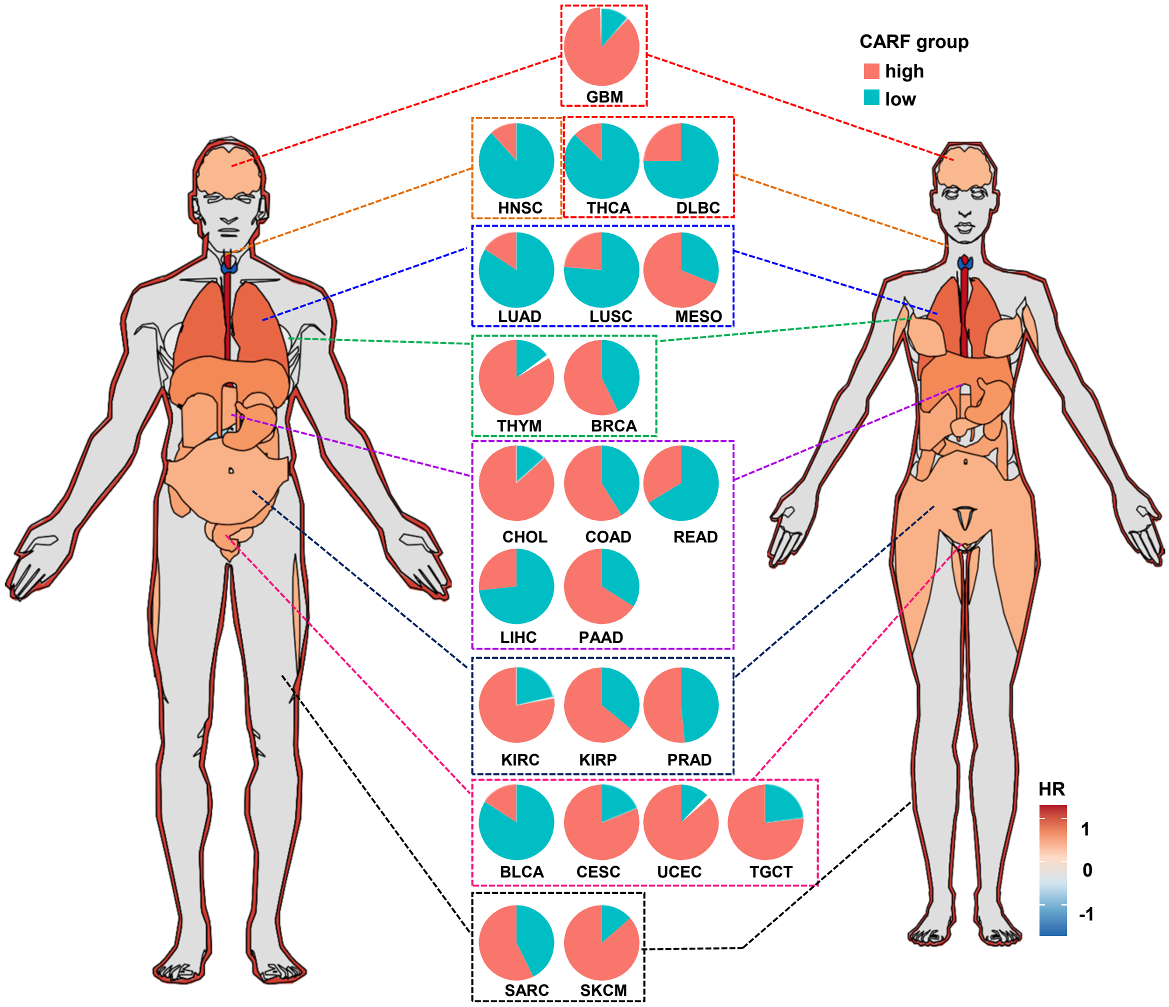

Figure S1. Anatogram plot of hazard ratios (HRs) for different tumor types grouped by high or low *CARF* expression, excluding DLBC. The color gradient reflects increasing HRs, with darker shades indicating higher risk for both male (left) and female (right) groups. The central pie chart shows the distribution of each tumor type categorized by high or low *CARF* expression. The abbreviations of cancer types are the same as in Figure 1B.

Qu et al., Supp Figure 2

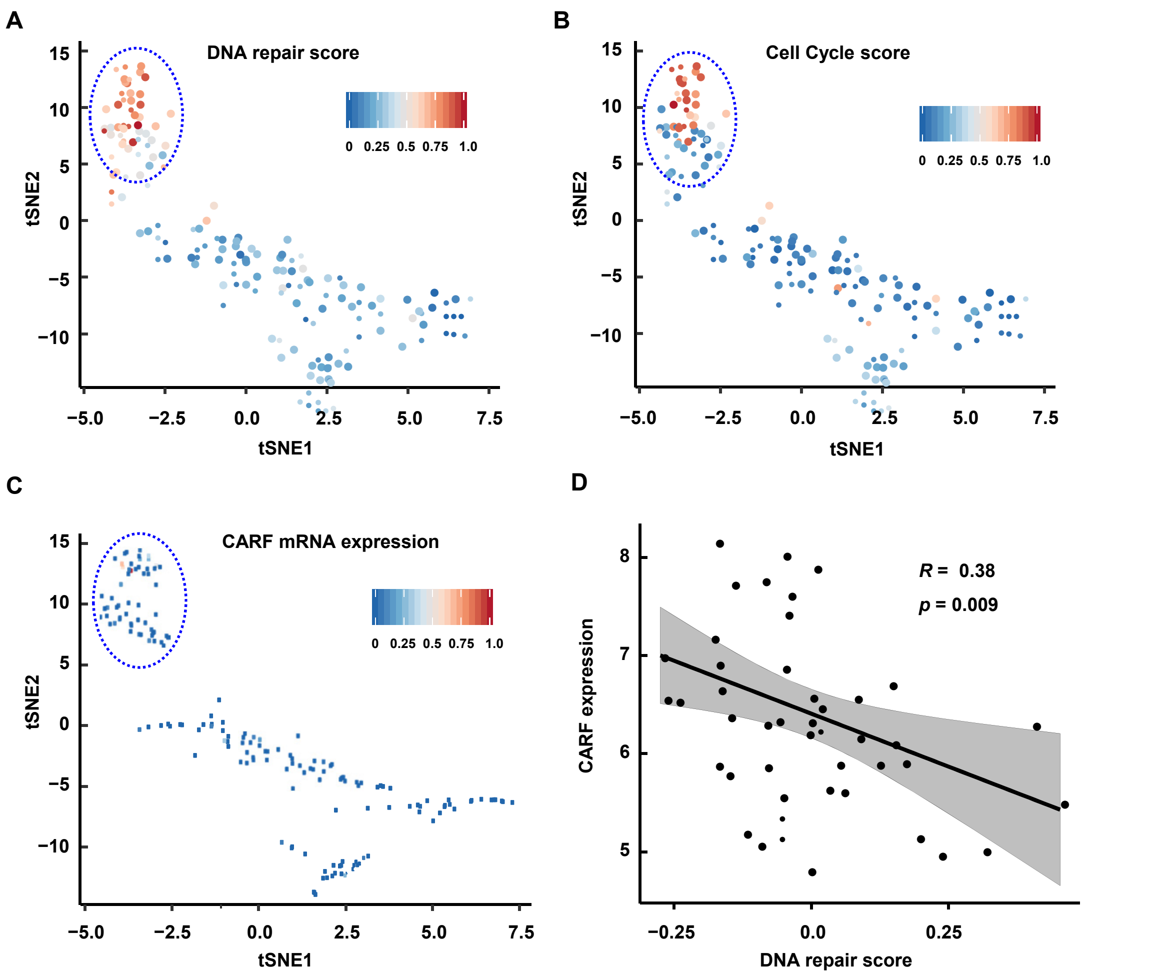

Figure S2. ***CARF*** **expression is associated with p53-related DNA repair and cell cycle regulation and inversely correlated with DNA repair in single-cell dataset.**

A-C. TSNE dimensionality reduction map of GSE110499 single-cell mRNA sequencing dataset from acute leukemia (AML). Color scale of points is GSVA enrichment score for DNA repair (A), cell cycle (B), or the normalized *CARF* mRNA expression values (C). tSNE1 or tSNE2 indicated two dimensions of projection.

D. Correlation analysis showed that *CARF* expression is closely related to DNA repair score. R is the correlation coefficient and p is the significance.

Qu et al., Supp Figure 3

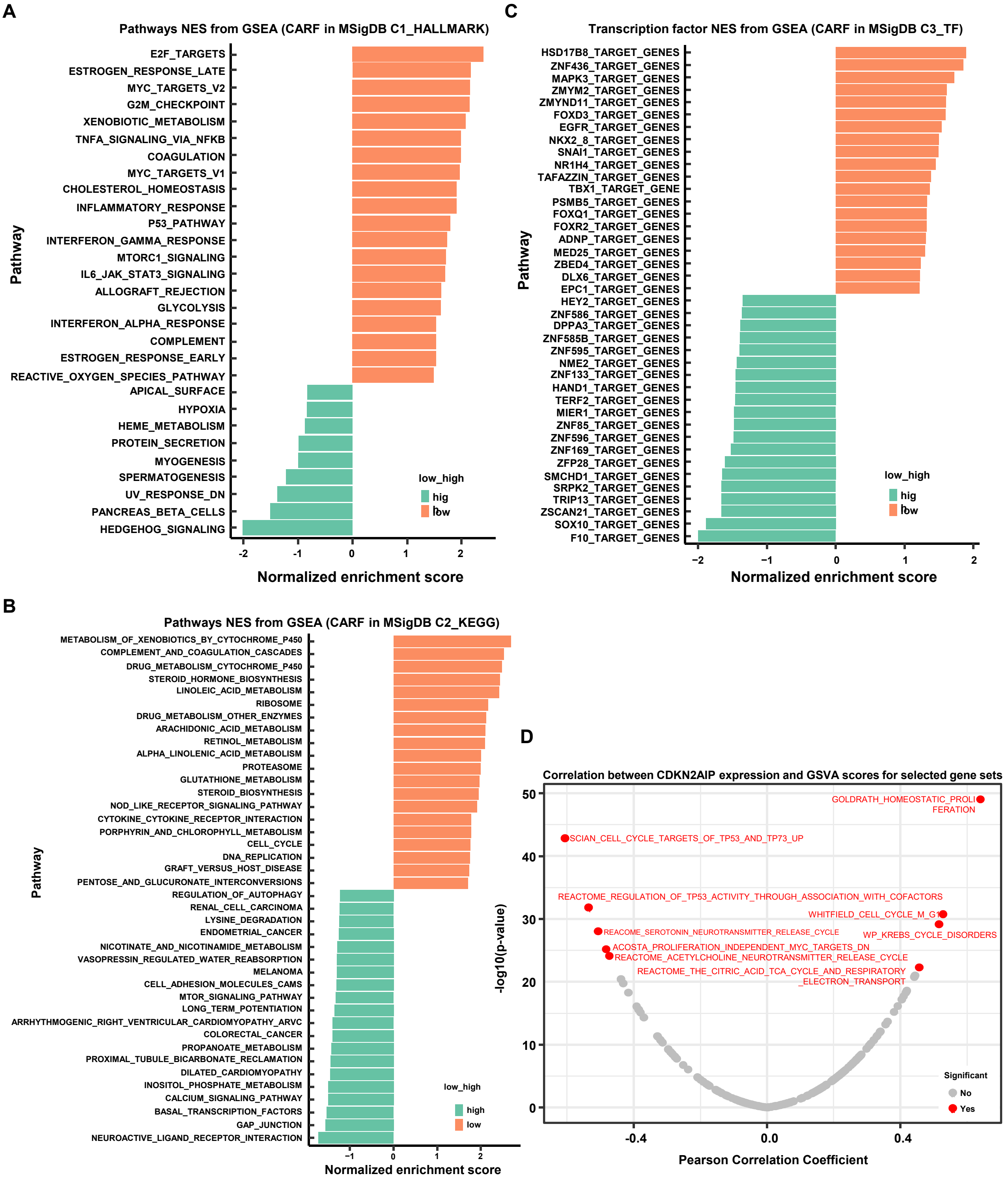

Figure S3. Biological processes enriched in tumor patients with high or low *CARF* expressions from TCGA database.

Gene Set Enrichment Analysis (GSEA) of hallmark genes and *CARF* expression. Mutsig C1 Hallmark dataset (A), Kyoto Encyclopedia of Genes and Genomes (KEGG) dataset (B), and Mutsig C3 transcription factor dataset (C) are shown. Bars represent the Normalized Enrichment Score (NES), quantifying the strength and direction of gene set enrichment.

D. Volcano Plot illustrates the correlation between CARF expression and GSVA enrichment scores for gene sets in Lymphoma (GSE10946). Each dot represents a gene set from the Mutsig database related to cell proliferation, cell cycle, or p53 pathway. The *x*-axis shows the Pearson correlation between CARF expression and gene set's GSVA score. Gene sets meeting significance ($p < 0.05$ and a preset correlation threshold) are highlighted and labeled. Positively correlated gene sets are mainly linked to cell proliferation and cell cycle, whereas negatively correlated ones are associated with p53 pathway, which is consistent with that higher *CARF* expression is associated with increased proliferation and reduced p53 activity in lymphoma.

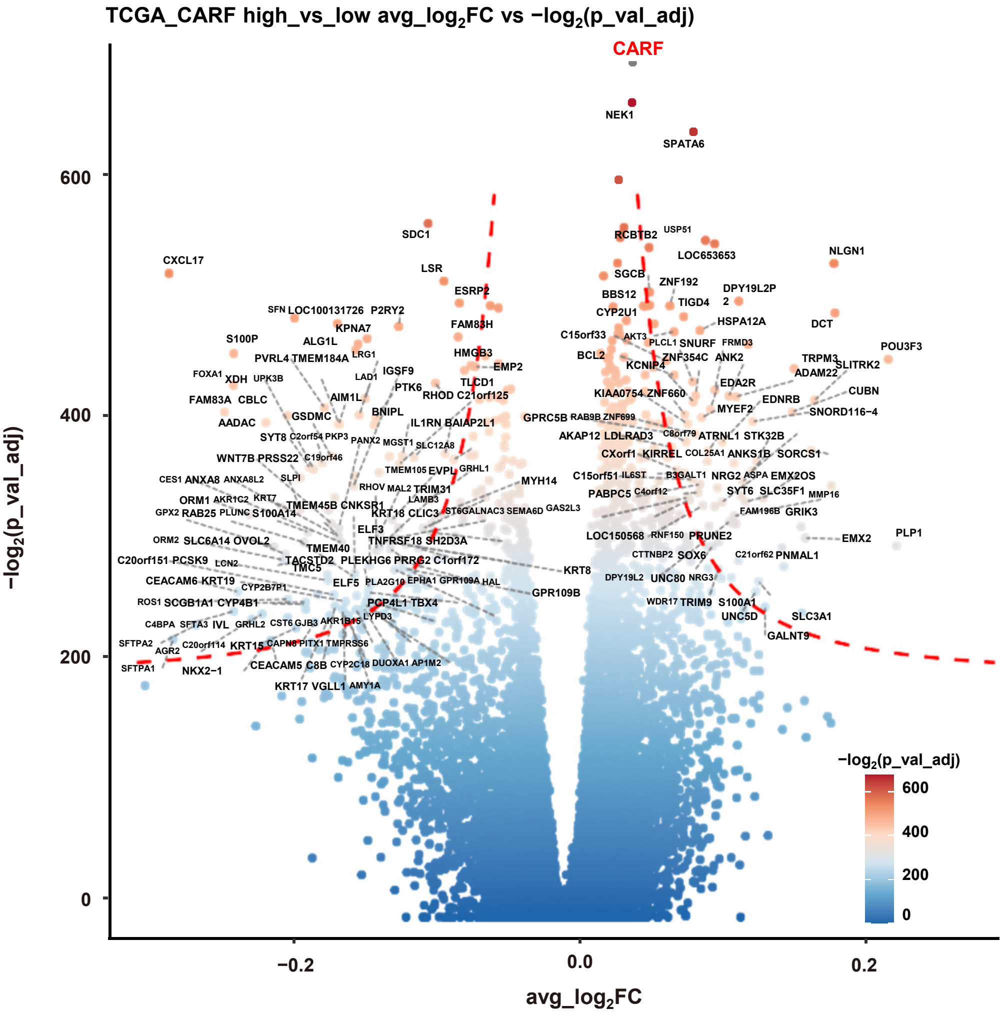

Figure S4. Volcano plot shows the differential *CARF* expression in TCGA database. The *x*-axis represents log2 fold change, indicating the magnitude of gene expression differences, and *y*-axis shows the log2(*p*-value), reflecting the statistical significance. The hyperbolic curve serves as a dynamic threshold that balances the statistical significance and biological relevance. Genes above the curve meet the criteria for both sufficient expression change and robust statistical significance, and are considered significantly differentially expressed. This method ensures the selection of genes with meaningful biological and statistical impact, reducing noise and avoiding the arbitrary cutoff biases.

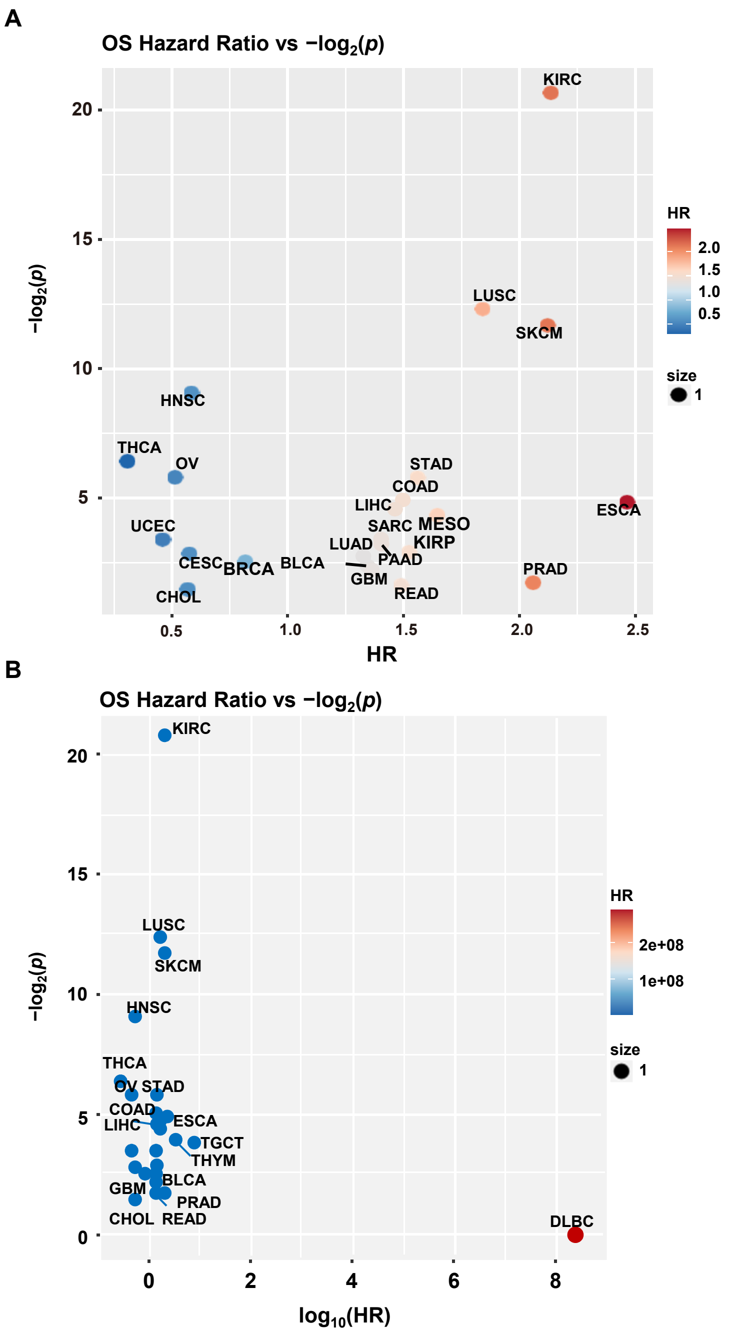

Figure S5. Volcano plots show the survival analysis of high or low *CARF* expression in different cancers with (A) or without (B) DLBC. Hazard Ratio (*x*-axis) and *p*-value (*y*-axis) for high and low *CARF* expression with (A) and without (B) DLBC were shown.

Qu et al., Supp Figure 6

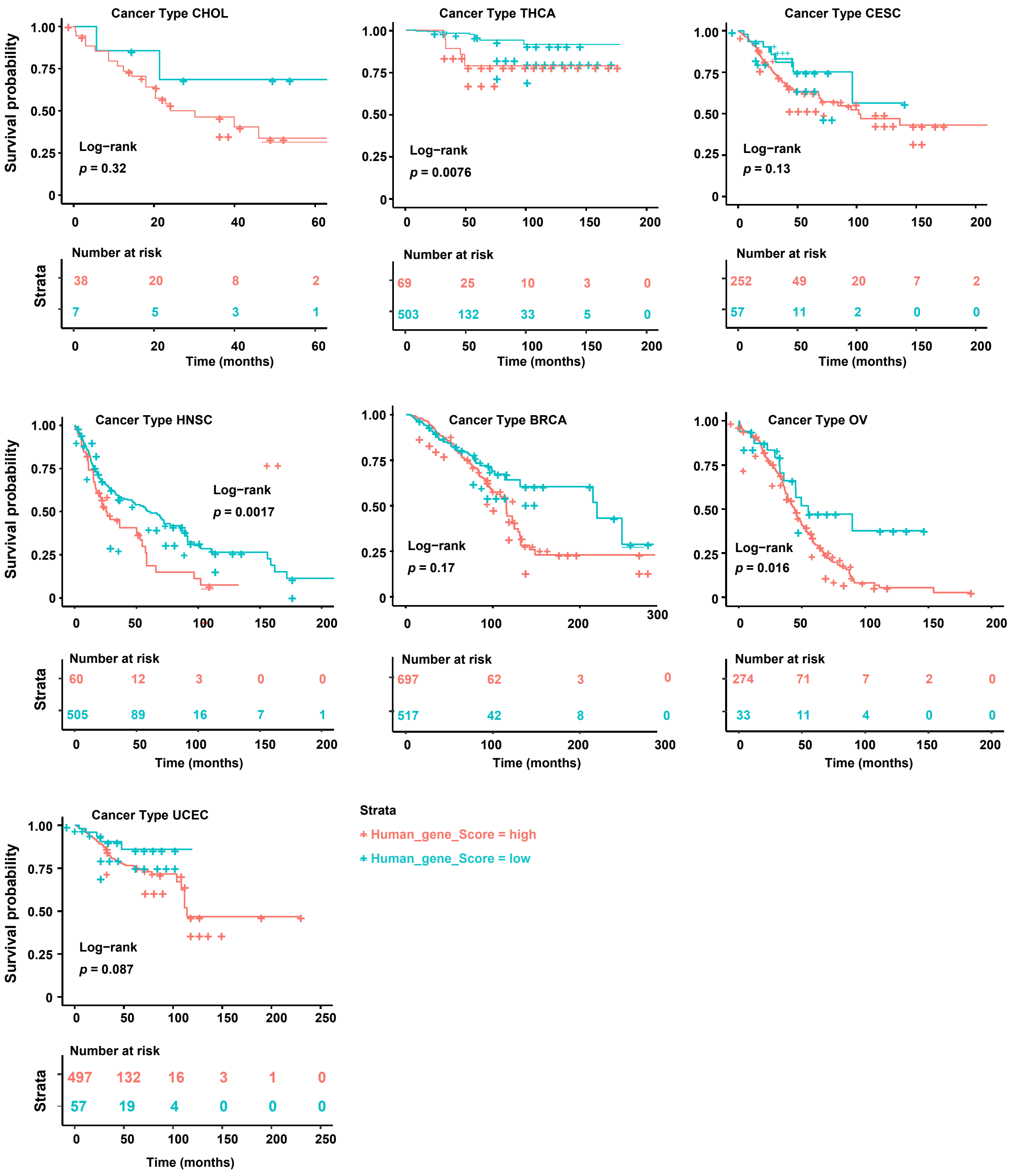

Figure S6. A pan-cancer analysis of *CARF* gene in TCGA database with HR <= 1. Survival curves for high and low *CARF* expression in different cancers with HR <= 1.

Qu et al., Supp Figure 7

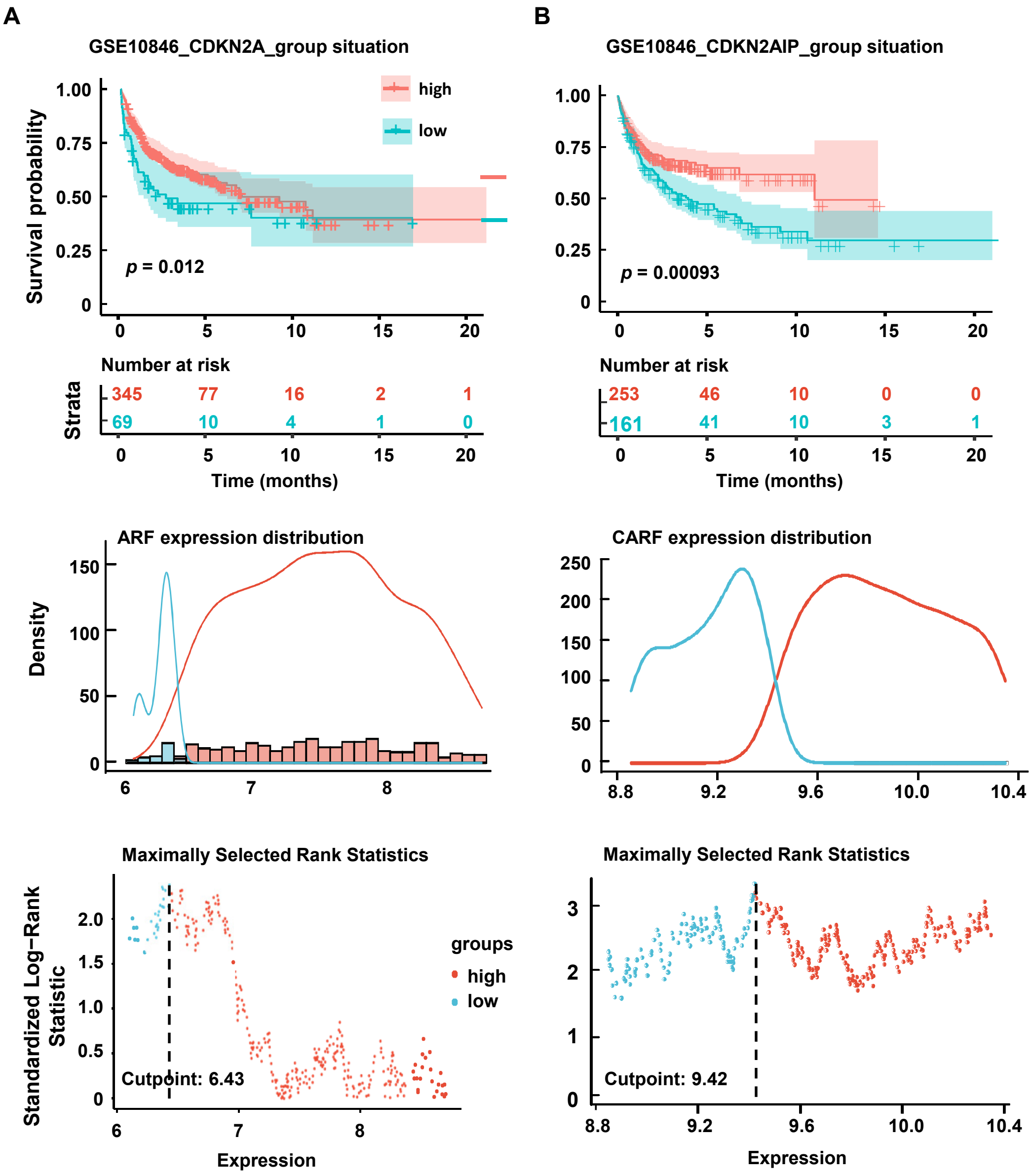

Figure S7. Grouping and survival analysis of *ARF* (A, *CDKN2A*) and *CARF* (B, *CDKN2AIP*) in Lymphoma GSE1084 database show that *CARF* is closely related to lymphoma. High and low expression groups were defined based on the minimum *p*-value cutoff, and final cutoffs optimized through result-oriented methods. Expression level distributions (middle) and standardized Log-Rank Statistic scatter plots (bottom) are shown. Standardized Log-Rank Statistic is a measure used in survival analysis, showing the probability of survival or the occurrence of events in individuals over a certain period of time.

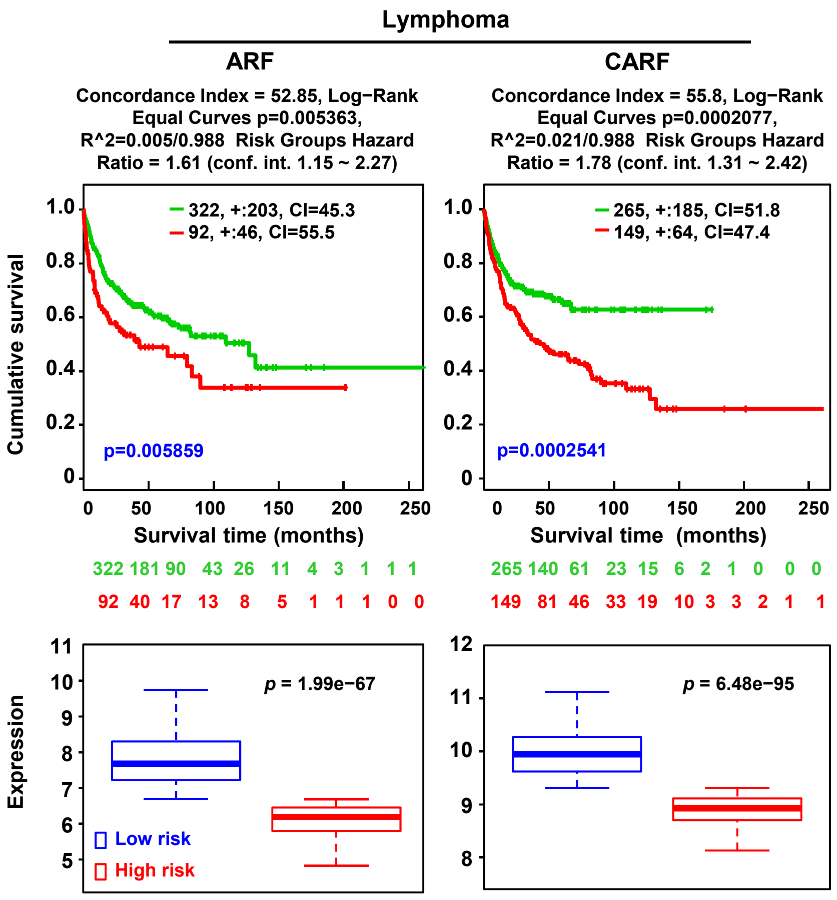

Figure S8. Survival analysis of *CARF* or *ARF* transcription levels in lymphoma patients. Cancer prognosis analysis showed a strong correlation between *CARF* or *ARF* transcription levels and prognosis in lymphoma patients. Low (indicated by green) or high (indicated by red) *CARF* expression was associated with worse or better prognosis, as determined using the SurvivaX tool (http://bioinformatica.mty.itesm.mx:8080/Biomatec/SurvivaX.jsp) and the Lenz Staudt Lymphoma GSE10846 database including prognostic information of 420 patients.

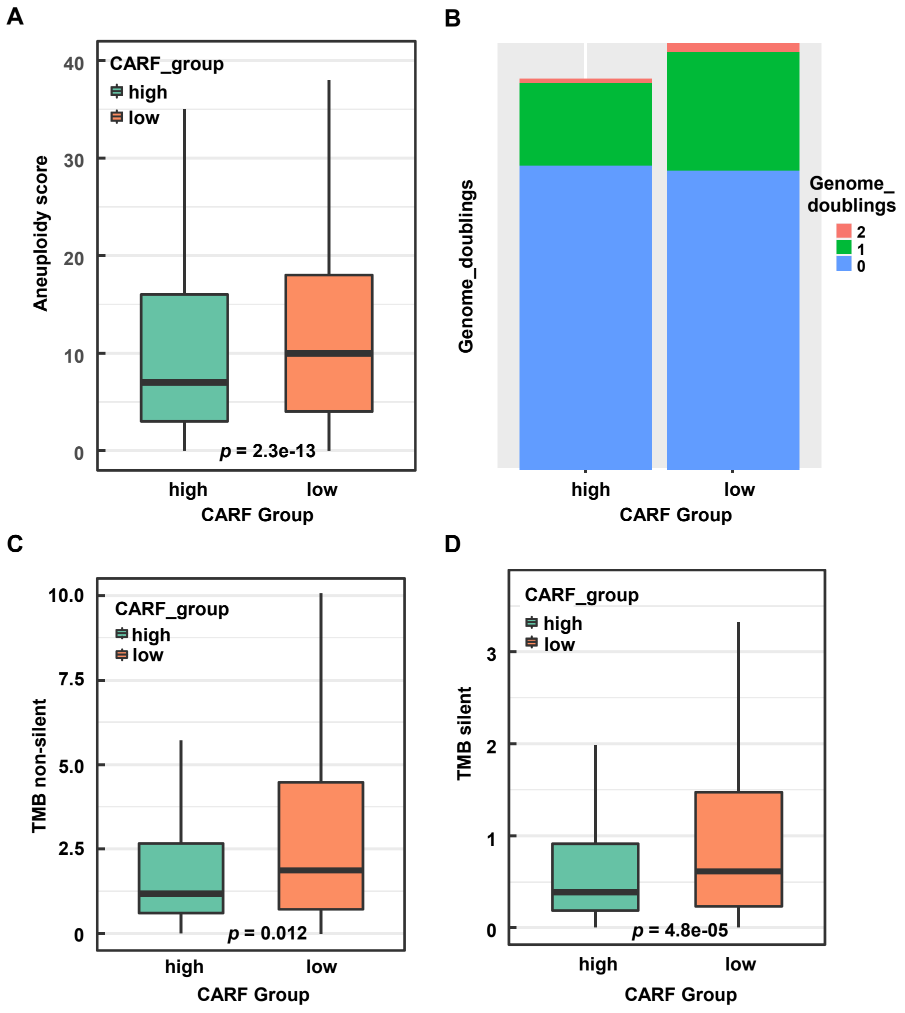

Figure S9. Analysis of *CARF* gene in Lymphoma GSE1084 database.

A. Aneuploidy Score, a metric for chromosomal abnormalities in a sample, indicates the extent of aneuploidy.

B. Genome doublings, representing the occurrence of chromosomal duplication in a cell or organism.

C. TMB non-silent, the number of non-synonymous mutations per megabase in a tumor genome, indicates the presence of functional alterations ($p < 0.05$).

D. TMB silent, the number of synonymous mutations per megabase in a tumor genome, indicates no functional alteration ($p < 0.001$).

Qu et al., Supp Figure 10

Variable		N	Hazard ratio		p
**R_CARF**	R_HC	198		Reference	
	NR_LC	126		2.07 (1.44, 2.96)	<0.001
**CARF_group**	high	253		Reference	
	low	161		1.67 (1.23, 2.27)	0.001
**age_group**	Age > 60 yr	179		Reference	
	Age >= 60 yr	235		2.08 (1.49, 2.90)	<0.001
**gender**	female	172		Reference	
	male	224		1.02 (0.74, 1.40)	0.897
**pathological_type**	Unclassified DLBCL	64		Reference	
	GCB DLBCL	183		0.72 (0.44, 1.19)	0.200
	ABC DLBCL	167		1.98 (1.24, 3.15)	0.004
**Ann_Arbor_stage**	Ann Arbor stage = I	66		Reference	
	Ann Arbor stage > II	348		4.06 (2.07, 7.95)	<0.001
**CHOP_status**	R−CHOP−Like Regimen	233		Reference	
	CHOP−Like Regimen	181		1.92 (1.39, 2.66)	<0.001
**extranodal_sites**	extranodal_sites < = 1	353		Reference	
	extranodal_sites > 1	30		1.93 (1.15, 3.25)	0.013
**ECOG_performance**	ECOG_performance < = 1	296		Reference	
	ECOG_performance > 1	93		2.84 (2.05, 3.92)	<0.001
**LDH**	LDH < = 1X	173		Reference	
	LDH > 1X normal	178		2.67 (1.87, 3.81)	<0.001

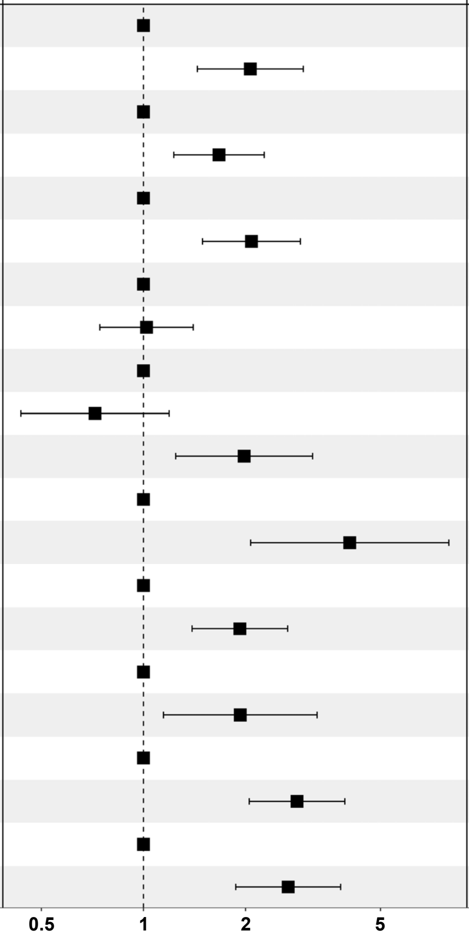

Figure S10. Univariate analysis of *CARF* expression and clinicopathological features in lymphoma GSE10846 cohort.

Qu et al., Supp Figure 11

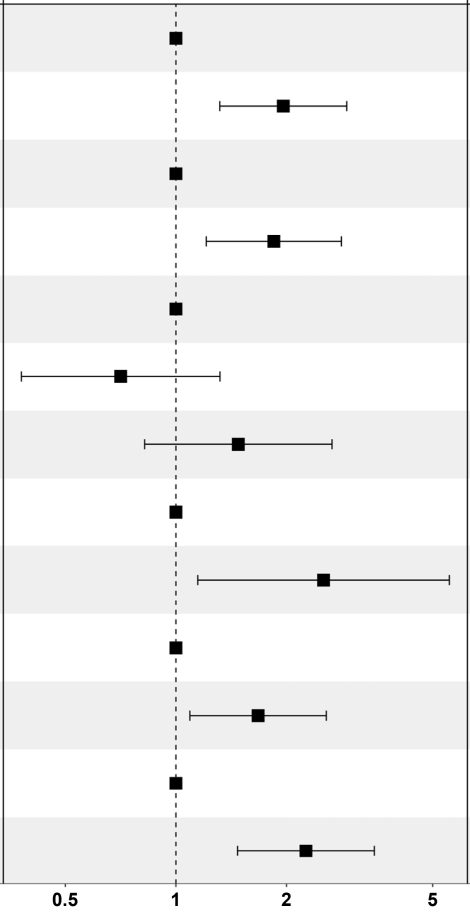

Variable		N	Hazard ratio	p
**R_CARF**	R_HC	157	Reference	
	NR_LC	112	1.96 (1.32, 2.92)	<0.001
**age_group**	Age > 60 yr	116	Reference	
	Age >= 60 yr	153	1.85 (1.21, 2.82)	0.005
**pathological_type**	Unclassified DLBCL	39	Reference	
	GCB DLBCL	122	0.71 (0.38, 1.32)	0.275
	ABC DLBCL	108	1.48 (0.82, 2.66)	0.192
**Ann_Arbor_stage**	Ann Arbor stage = I	44	Reference	
	Ann Arbor stage > II	225	2.52 (1.15, 5.55)	0.021
**ECOG_performance**	ECOG_performance < = 1	206	Reference	
	ECOG_performance > 1	63	1.67 (1.09, 2.57)	0.018
**LDH**	LDH < = 1X	139	Reference	
	LDH > 1X normal	130	2.26 (1.47, 3.47)	<0.001

Figure S11. Multivariate analysis of *CARF* expression groups and clinicopathological features in lymphoma GSE10846 cohort.

Qu et al., Supp Figure 12

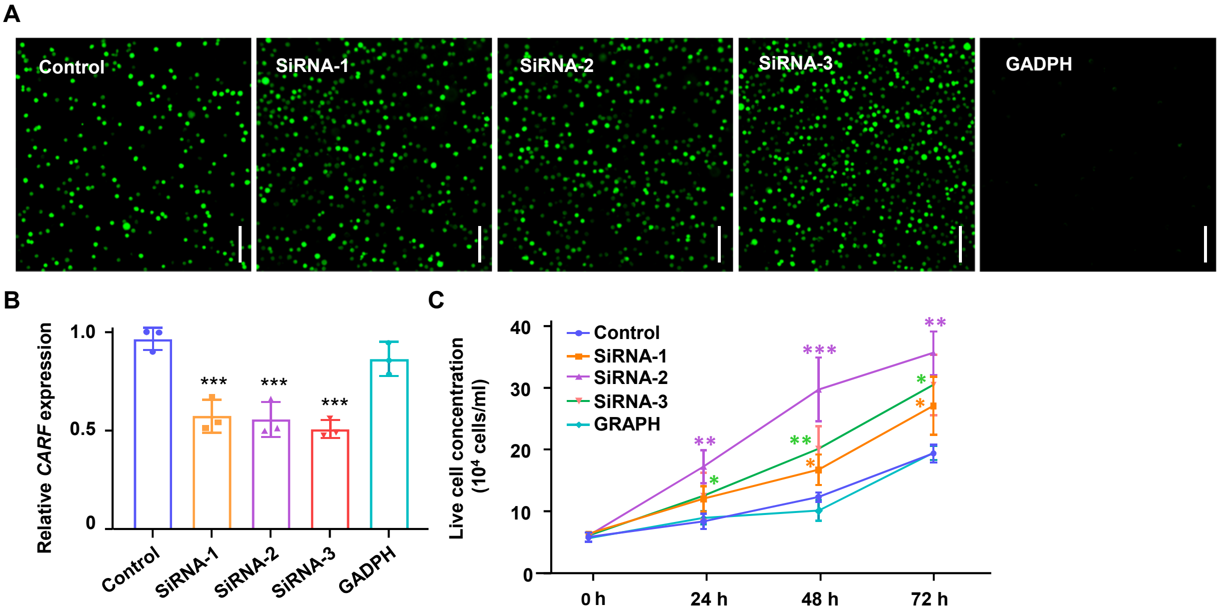

Figure S12. siRNA of *CARF* promotes cell division in Burkitt's (Daudi) lymphoma cell lines.

A. Visualization of tagged CARF-siRNA-GFP in Burkitt's (Daudi) lymphoma cells shows that *CARF*-siRNA promotes cell division. Bar = 100 μm.

B. qPCR analysis of *CARF* expression in different siRNA transfection lines. Experiments were biologically repeated three times and data were means ± SD (n = 3). Statistical significance is determined by using one-way ANOVA (***, $p < 0.001$, compared with vector transfection).

C. Live cell concentrations of cell lines at 0, 24, 48 or 72 h post-transfection. Experiments were biologically repeated three times and data were means ± SD (n = 5). Statistical significance is determined by using one-way ANOVA (*, $p < 0.05$; **, $p < 0.01$; ***, $p < 0.001$, compared with vector transfection).

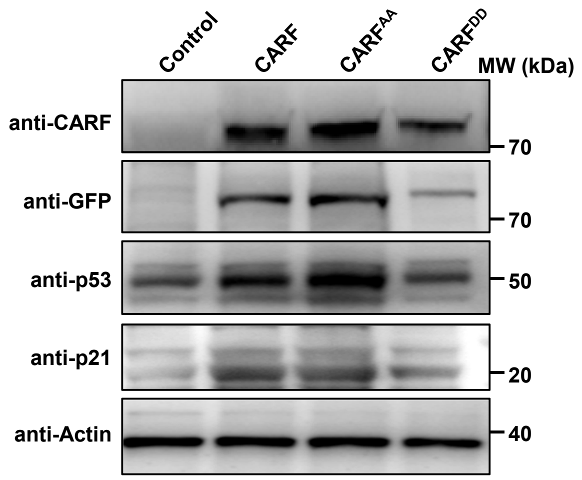

Figure S13. Western blotting analysis of CARF, p53 and p21 proteins in RA1 lymphoma cell lines 6 days post-transfection. Constructs expressing *CARF*, $CARF^{AA}$ and $CARF^{DD}$ were transfected into RA1 lymphoma cell lines, respectively. Actin was used as a loading control.

Qu et al., Supp Fig. 14

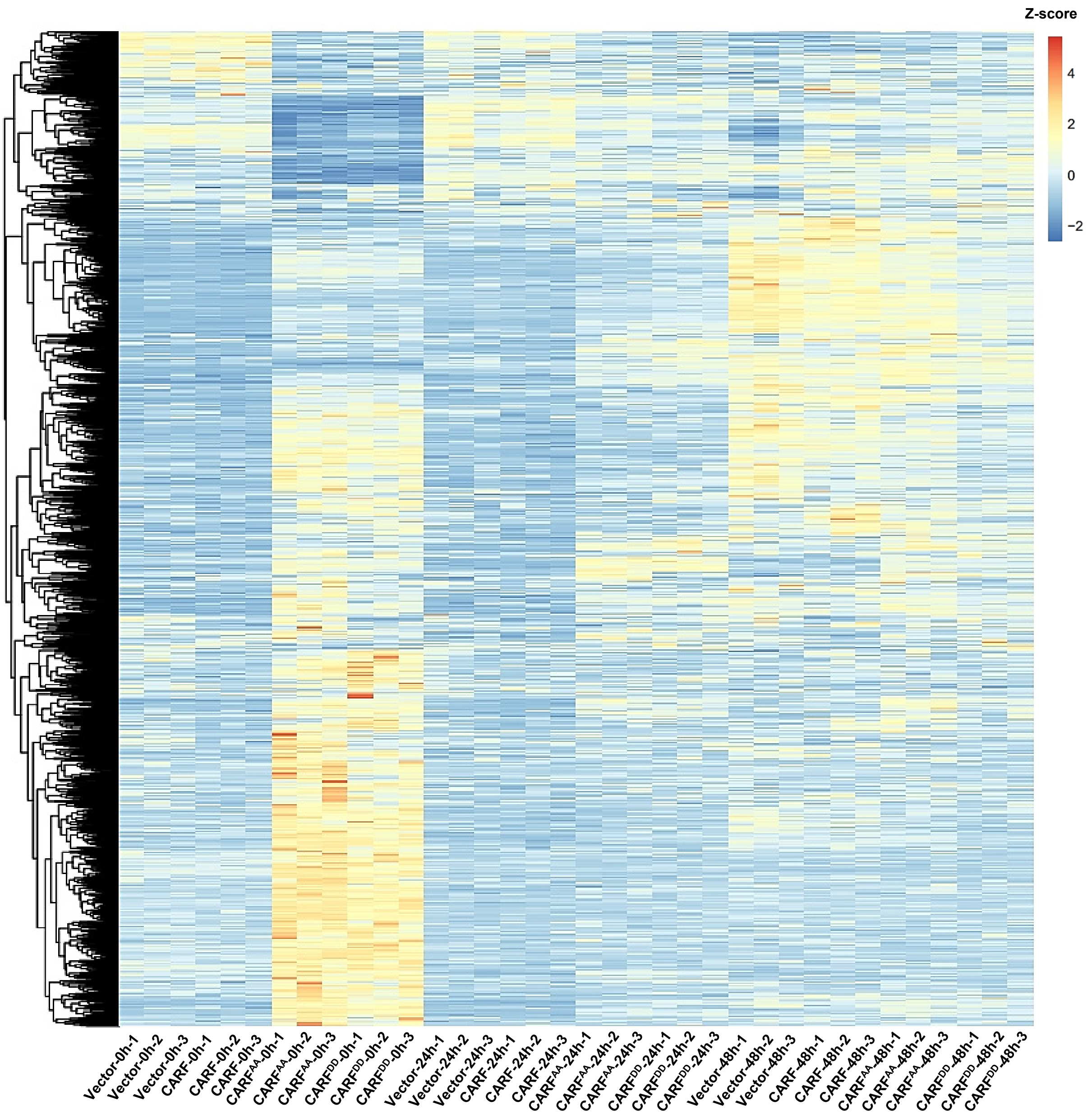

Figure S14. Heatmap of the differentially expressed genes (DEGs) from RNA-seq analysis.

Heatmap of all DEGs in transcriptome sequencing (RNA-seq) analysis with |log2FC| > 0.5, adjusted $p < 0.05$. RNA of RA1 lymphoma cells expressing *CARF*, $CARF^{AA}$, $CARF^{DD}$ at 0, 24 and 48 h were extracted and used for sequencing. Experiments were biologically repeated three times. Z-score was calculated with log2-transformed FPKM values.

A

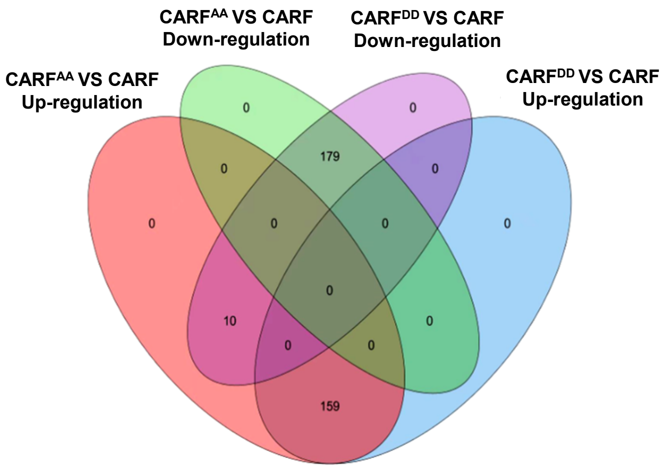

B

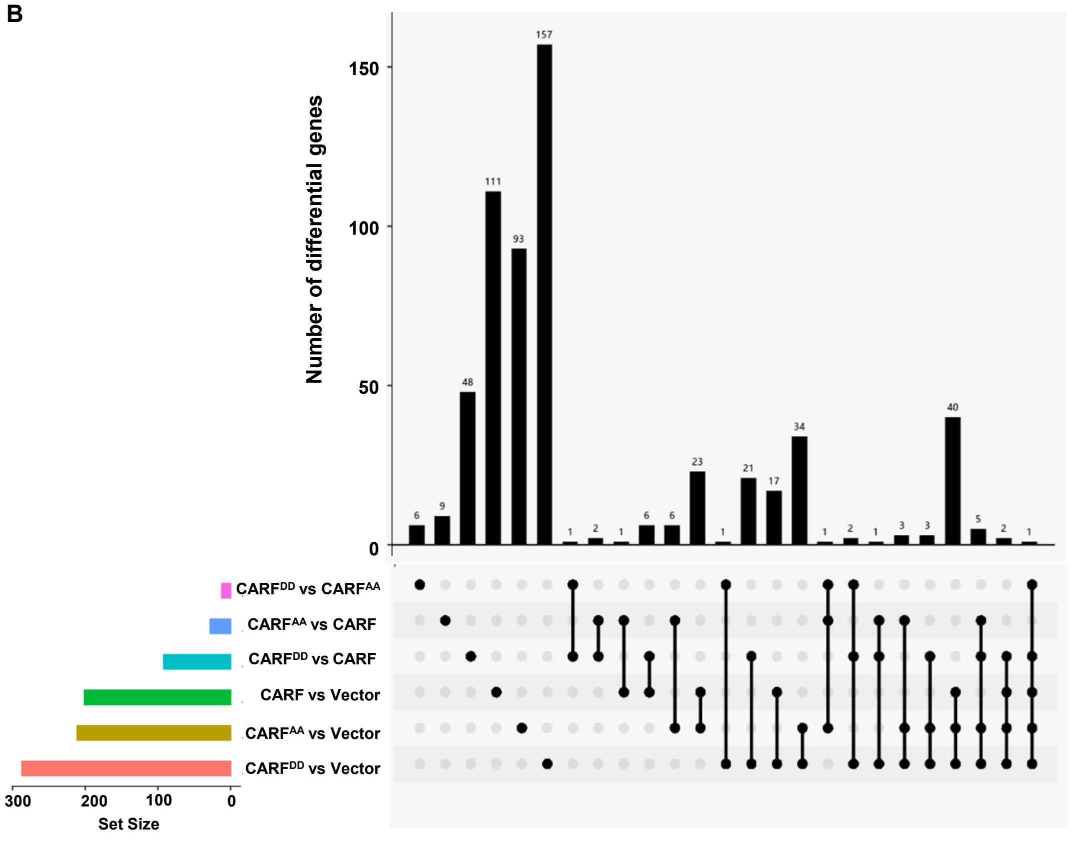

Figure S15. RNA-seq analysis shows the differential gene expression in CARF variants with mutated phosphorylation sites.

A. Venn diagrams representing the overlapped upregulated and downregulated genes in RA1 cells expressing *CARF*^{AA} or *CARF*^{DD} compared to CARF (|log2FC| > 0.5, adjusted $p < 0.05$). Experiments were biologically repeated for three times.

B. Distribution and number of DEGs in RA1 cells expressing *CARF*^{AA} or *CARF*^{DD} compared to *CARF* or vector (|log2FC| > 0.5, adjusted $p < 0.05$).

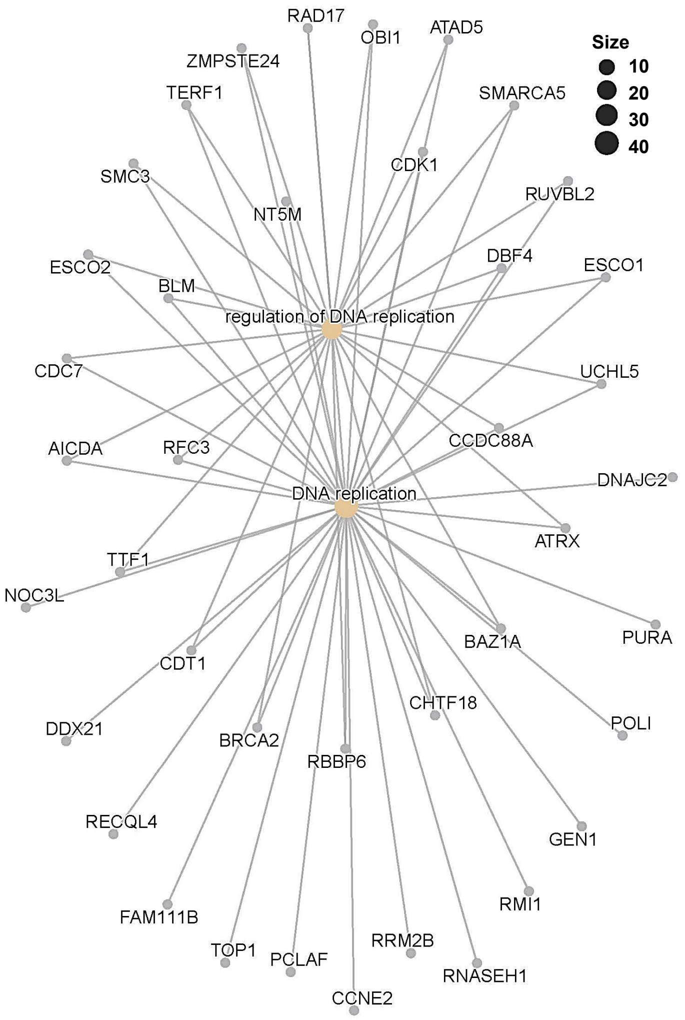

Figure S16. **Functional enrichment analysis of DEGs related to DNA replication regulation.** Size of dots reflects the number of genes.

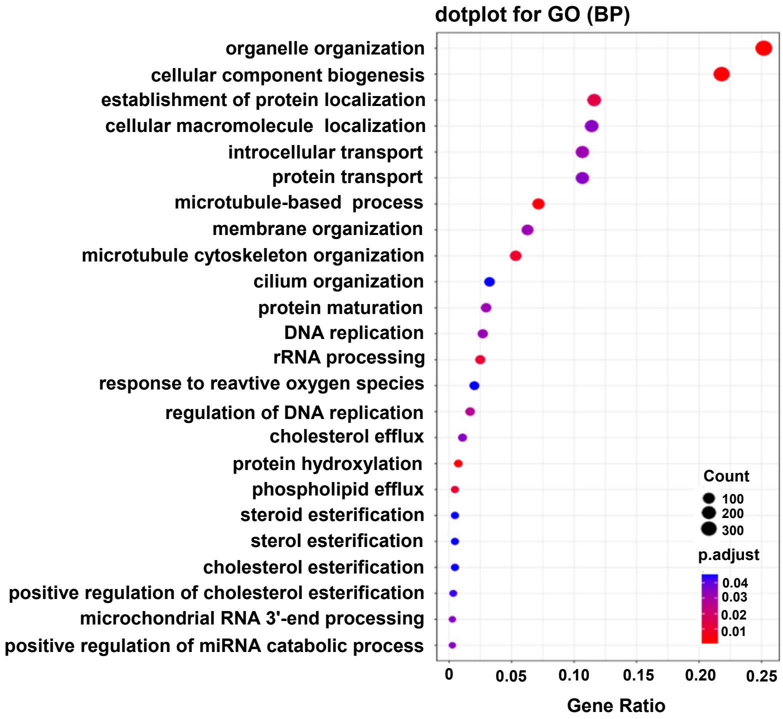

Figure S17. **Molecular functions of DEGs in the transcriptome sequencing analysis.** The top 25 GO categories are shown in descending order (right) with statistical significance (|log2FC| > 0.5, adjusted $p < 0.05$).

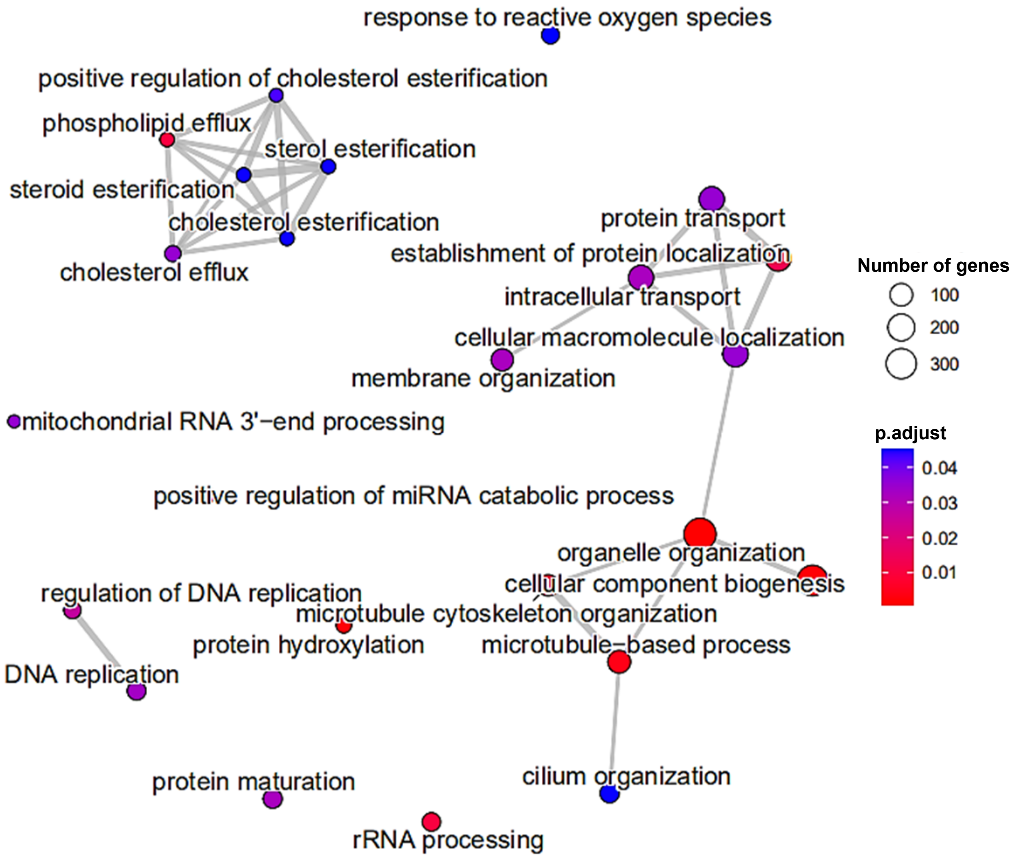

Figure S18. Molecular function analysis of DEGs shows the crucial roles of phosphorylation sites of CARF in cell cycle regulation.

Enrichment analysis of molecular functions of DEGs in RA1 cells expressing $CARF^{AA}$ or $CARF^{DD}$ compared to *CARF* revealed the enriched cell cycle regulation related processes including DNA replication, microtubule-based process and protein transport and localization.

Qu et al., Supp Figure 19

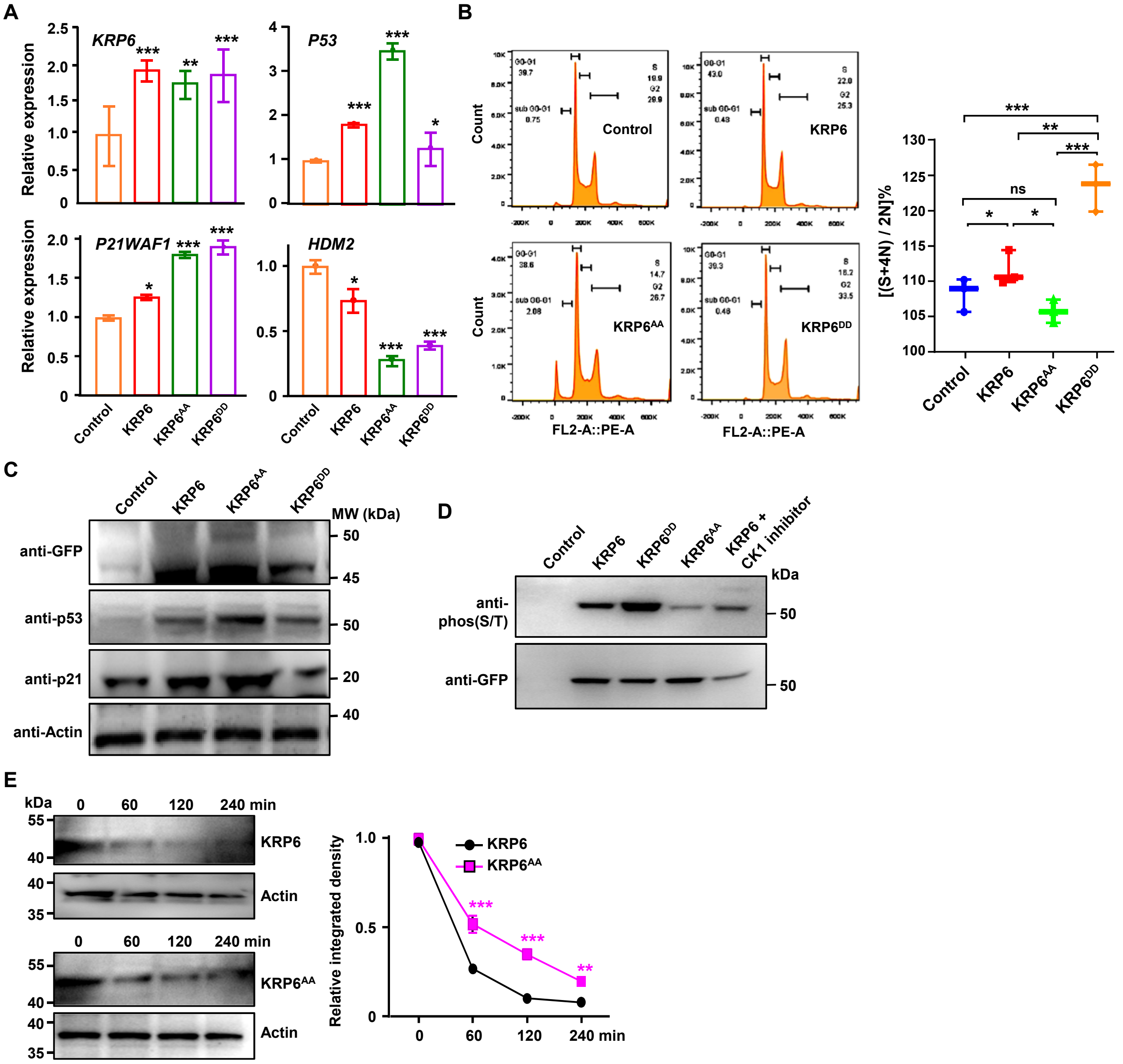

Figure S19. Non-phosphorylation variant of *Arabidopsis* KRP6 inhibits lymphoma formation and growth.

A. qPCR analysis of *KRP6*, *P53*, *P21WAF1*, and *HDM2* expression in RA1 transfection cells. Experiments were biologically repeated three times, and data were means ± SD (n = 3). Statistical significance was determined by using one-way ANOVA (*, $p < 0.05$; **, $p < 0.01$; ***, $p < 0.001$, compared to vector transfection).

B. Cytometry analysis showed that KRP6^{AA} significantly suppresses the cell division in RA1 lymphoma cells. Cells were digested into single cells at 96 h post-transfection for cell division analysis by flow cytometry (left). "FL2-A::PE-A" represents a detection channel and the fluorescent dye used in the assay. Proportions of cells with different DNA content were calculated and data were shown as means ± SD (n = 3, right). Statistical significance was analyzed by using Tukey's multiple comparisons test following one-way ANOVA (*, $p < 0.05$; **, $p < 0.01$; ***, $p < 0.001$; ns, no significance).

C. Western blotting analysis of KRP6, p53 and p21 protein levels in RA1 transfection cells. Recombinant KRP6 protein or variants were co-expressed with EGFP and examined using anti-GFP antibodies. Proteins p53 and p21 were examined using anti-p53 or anti-p21 antibodies. Actin was used as a loading control.

D. *In vivo* phosphorylation assay showed that phosphorylation levels of cells expressing *KRP6^{AA}* or *KRP6* with CK1 inhibitor reduced significantly. Burkitt's Ramos (RA1) lymphoma cells expressing *KRP6*, *KRP6^{AA}* and *KRP6^{DD}* fused with EGFP were used. Anti-phos (S/T) indicates the phosphorylation signals and anti-GFP antibody confirms the protein loading.

E. Western blotting analysis confirms the suppressed degradation of non-phosphorylation variant KRP6^{AA} in RA1 transfection cells. Stability of KRP6-EGFP and KRP6^{AA}-EGFP fusion proteins was examined with anti-GFP antibody (left, Actin protein was analyzed with anti-Actin antibody and used as loading control). Band density (right) was measured by Image J, and relative density was normalized to KRP6 (or KRP6^{AA}) intensity at time 0, which was set as 1.0. Data were means ± SD (n = 3), and statistical significance was determined by using one-way ANOVA (**, $p < 0.01$; ***, $p < 0.001$).
